# HIMF deletion ameliorates acute myocardial ischemic injury by promoting macrophage transformation to reparative subtype

**DOI:** 10.1007/s00395-021-00867-7

**Published:** 2021-04-23

**Authors:** Yanjiao Li, Min Dong, Qing Wang, Santosh Kumar, Rui Zhang, Wanwen Cheng, Jiaqing Xiang, Gang Wang, Kunfu Ouyang, Ruxing Zhou, Yaohong Xie, Yishen Lu, Jing Yi, Haixia Duan, Jie Liu

**Affiliations:** 1 Guangdong Key Laboratory of Genome Stability and Human Disease Prevention, Department of Pathophysiology, Shenzhen University Health Science Center, Shenzhen, 518060 China; 2 Guangdong Key Laboratory of Regional Immunity and Diseases, Department of Pathology, Shenzhen University Health Science Center, Shenzhen, 518060 China; 3 Drug Discovery Center, State Key Laboratory of Chemical Oncogenomics, School of Chemical Biology and Biotechnology, Shenzhen Graduate School, Peking University, Shenzhen, 51055 China

**Keywords:** Myocardial infarction, Macrophage polarization, Hypoxia-induced mitogenic factor, Tissue repair, Cardiac fibroblast, C/EBP-homologous protein

## Abstract

Appropriately manipulating macrophage M1/M2 phenotypic transition is a promising therapeutic strategy for tissue repair after myocardial infarction (MI). Here we showed that gene ablation of hypoxia-induced mitogenic factor (HIMF) in mice (*Himf*^*−/−*^ and HIMF^flox/flox^;Lyz2-Cre) attenuated M1 macrophage-dominated inflammatory response and promoted M2 macrophage accumulation in infarcted hearts. This in turn reduced myocardial infarct size and improved cardiac function after MI. Correspondingly, expression of HIMF in macrophages induced expression of pro-inflammatory cytokines; the culturing medium of HIMF-overexpressing macrophages impaired the cardiac fibroblast viability and function. Furthermore, macrophage HIMF was found to up-regulate C/EBP-homologous protein (CHOP) expression, which exaggerated the release of pro-inflammatory cytokines via activating signal transducer of activator of transcription 1 (STAT1) and 3 (STAT3) signaling. Together these data suggested that HIMF promotes M1-type and prohibits M2-type macrophage polarization by activating the CHOP–STAT1/STAT3 signaling pathway to negatively regulate myocardial repair. HIMF might thus constitute a novel target to treat MI.

**Supplementary Information:**

The online version contains supplementary material available at 10.1007/s00395-021-00867-7.

## Introduction

Inflammation and cytokine elaboration promotes the clearance of damaged tissue and have active roles in left ventricular (LV) remodeling after myocardial infarction (MI) [31]. However, when the inflammatory response is overheated, it exaggerates infarct expansion and prohibits wound repair, leading to adverse LV remodeling and long-term heart failure [6, 21, 41]. Continuous efforts are being made to modulate MI-induced inflammation [6, 26]. Macrophages are the predominant immune cell type to infiltrate the infarcted myocardium. They orchestrate the inflammatory response by polarizing into distinct pro-inflammatory (Ly6C^high^, M1-like) or pro-healing (Ly6C^low^, M2-like) subpopulations [22, 23]. Initial ischemic injury triggers macrophage infiltration and M1 polarization in the first 3 days post MI [23]. These cells display phagocytic and pro-inflammatory properties, highly express pro-inflammatory genes [e.g. interleukin-1β, inducible nitric oxide synthase (NOS2)], meanwhile release cytotoxic molecules [e.g. nitric oxide, reactive oxygen species], to clear damaged myocardial cells and set the stage for wound healing [40]. Besides, exosomes derived from M1 inflammatory macrophage were found to suppress angiogenesis and exacerbate cardiac dysfunction post MI [19]. Around 4–7 days post MI, M2 macrophages increase in number and become the predominant cell type. M2 macrophages express anti-inflammatory cytokines, such as IL-10 that suppresses pro-inflammatory cytokine synthesis. They also express reparative cytokines and enzymes, such as arginase 1 (Arg1) and transforming growth factor (TGF) *β* to promote collagen and extracellular matrix protein deposition [3, 40]. It has been reported that transplantation of M2 reparative macrophage evidently improves the cardiac function recovery of MI [30]. Thus, the predominance and activity of M2 macrophages in the infarcted myocardium facilitates inflammation resolution and tissue repair. Despite advances in our understanding on the role of macrophages during MI repair, the mechanisms regulating M1/M2 phenotypic transformation are unclear.

Hypoxia-induced mitogenic factor (HIMF)—also known as resistin-like molecules (RELMα) or found in inflammatory zone (FIZZ1)—belongs to a cysteine-rich RELM family that is highly conserved in mammals [5]. There are four murine isoforms (i.e. HIMF/RELMα/FIZZ1, RELMβ/FIZZ2, Resistin/FIZZ3, and RELMγ/FIZZ4) and two human isoforms (i.e. RELMβ/FIZZ2, Resistin/FIZZ3). Each isoform shows unique tissue distribution and expression pattern, exerting different biological roles [37]. Strikingly, HIMF can be stimulated in immune cells [5, 13, 24, 27], especially in macrophages where HIMF is highly inducible by T helper (Th) 2 cytokines. HIMF is thus considered as an M2 macrophage biomarker, despite its function in macrophages is largely unknown [32]. Previous studies found distinct roles of HIMF in the immune response during different pathologies. For example, HIMF expression suppresses Th2 immune responses in mouse models of helminth infection and Schistosoma mansoni egg challenge [24]; while it has no effect on the Th2 response yet promotes the Th17 response in a mouse model challenged with intermittent ovalbumin and a colitis model induced by bacterial infection [5, 27]. As Th17 cytokines are pro-inflammatory and Th2 cytokines mediate tissue remodeling and fibrosis [1, 28], these immune responses share functional similarities with the respective M1- and M2-macrophage-dominated responses after MI. Thus, it is intriguing to know whether HIMF participates in macrophage transformation and its possible role in modulating the inflammation response and tissue repairing during MI.

Here, we conducted our studies in *Himf*^*−/−*^ and WT mouse models of MI induced by coronary artery ligation, and compared the cardiac outcomes, M1/M2 macrophage transformation and M1/M2-related cytokines after MI. The in vivo outcomes were further examined in the macrophage-specific knockout mouse (HIMF^flox/flox^;Lyz2-Cre) and their littermate Flox mouse models of MI. The direct effect of HIMF on macrophage transformation and the underlying mechanisms were studied in cultured mouse bone marrow-derived macrophages (BMDMs) and RAW 264.7 murine macrophages. Furthermore, we investigated the crosstalk between HIMF-overexpressing macrophages and cardiac fibroblasts (CFs).

## Materials and methods

### Animal care

Animals were purchased from the Animal Center of Guangdong Province, China, and housed under pathogen-free conditions with free access to food and water. All animal procedures were approved by the Institutional Care and Ethical Committee of Shenzhen University, China and conformed to Guide for the Care and Use of Laboratory Animals (National Institutes of Health publication No. 85-23, revised 1996).

### Generation of the HIMF Knockout (***Himf***^***−/−***^) and HIMF^flox/flox^;Lyz2-Cre (***Himf***-CKO) mice

*Himf*^*−/−*^ mice were generated via a CRISP/Cas9 system, which resulted in the deletion of all four exons of HIMF gene, as previously described [14]. The genotyping was performed by PCR with 5′–GTGCTGATGCTGACTGTA–3′ and 5′–GATGACACTGCTTCCATAAG–3′ primers to identify the HIMF allele (504 bp) or 5′–CTCTTGAACCACACCTCTT–3′ and 5′–CTAACCAGGCATCTCACAT–3′ primers to identify the *Himf*^*−/−*^ allele (239 bp). *Himf*-CKO mice were generated using CRISP/Cas9/Cre method by Cyagen Biosciences (Guangzhou, China, Supplementary Fig. 1a). Briefly, the gRNA targeting HIMF gene, the donor vector-containing loxP sites, and Cas9 mRNA were co-injected into fertilized mouse eggs to generate HIMF-floxed offspring. The macrophage-specific deletion of HIMF was achieved by crossing HIMF-floxed mice with the Lyz2-Cre mice. The genotyping was performed by PCR with 5′–CTCTTCCTGTCTCTCAAGTGTCTGG–3′ and 5′–GGATCCTAACTGTTCGTTCTT CTT–3′ primers to identify the Flox insertion (334 bp, or 266 bp for WT band), or 5′–CCCAGAAATGCCAGATTACG–3′ and 5′–CTTGGGCTGCCAGAATTTCTC–3′ to identify the Cre allele (~ 700 bp) (Supplementary Fig. 1b).

### Model of myocardial infarction

Myocardial infarction (MI) was induced in C57BL/6 J male mice (10–12 weeks old, 25 ± 2 g) by permanent ligation of the left anterior descending coronary artery (LAD). The mice were anesthetized with 3% pentobarbital sodium, intubated and mechanically ventilated (100 strokes/min, 250 μL stroke volume, Hugo Sachs Elektronik-Harvard Apparatus). Each mouse was placed on a heating plate to maintain the body temperature, and a left thoracotomy was performed in the third left intercostal space to expose the heart. The LAD was ligated with a 7–0 silk suture at a depth of 1 mm and a width of 1–1.5 mm. The ischemia and whitening of the area between the ligation position and the heart apex suggested successful LAD ligation. The chest and skin were closed in layers with a 7–0 nylon suture and the air was removed from the thorax with a pleural catheter, followed by subcutaneous injection of 0.2 ml 0.9% saline for rehydration. For the sham operation, mice underwent the same procedure except for LAD ligation. The same surgeon blinded to genotypes performed MI and sham operations. The mice were sacrificed on day 3 post MI/sham operation to analyze the inflammatory phase, or on day 7 to analyze the transition from the inflammatory to the reparative phase. The left ventricle of MI mice was either collected as a whole sample or separated into the remote zone, border zone and infarcted zone according to the specific experiment aims. For comparison, left ventricle were collected from sham mice as a control for the surgical procedure. Tissue samples were immediately placed into TRIzol (Cat#15596018, Invitrogen, Carlsbad, CA) or RIPA lysis buffer (Cat#R0010, Solarbio, Beijing, China) for subsequent RNA or protein extraction, respectively, or snap frozen in liquid nitrogen and stored at − 80 °C for further processing. For sampling of different region of MI heart, we collected whitening papery region at 3 day and 7 day post MI as infarct zone, collected the border zone in 1 mm distance to the infarcted area from the left ventricle, collected the remote zone 2 mm away from the infarcted area from the left ventricle.

### Echocardiography

Echocardiography was performed on anesthetized mice (with 1.0% isoflurane) using a Vevo 2100 system (Visual Sonics, Toronto, Ontario, Canada), as previously described [15]. Briefly, the heart image was captured in the two-dimensional (2-D) mode in the parasternal short-axis view. M-mode tracings were recorded at the papillary muscle level and the following parameters were measured: the left ventricular (LV) internal dimensions at diastole (LVIDd) and systole (LVIDs), LV posterior wall dimensions at diastole (LVPWd) and systole (LVPWs). The LV fractional shortening (FS,  %) was calculated as [(LVIDd − LVIDs)/LVIDd] × 100, and the LV ejection fraction (EF, %) was calculated as [(LVIDd^2 ^− LVIDs^2^)/LVIDd^2^] × 100. An individual observer blinded to mice genotypes performed the echocardiography and subsequent data analysis.

### Flow cytometry

Mice were anesthetized with 3% pentobarbital sodium and the heart was quickly exposed and perfused with 10 ml sterile cold phosphate-buffered saline (PBS, Cat#AAPR52, PythonBio, Guangzhou, China). After removal, the heart was minced in 25 μl PBS, and digested in Dulbecco’s modified eagle’s medium (DMEM, Cat#C11995500BT, Gibco, Hyclone) solution with 60 U/ml hyaluronidase (Cat#H3506, Sigma-Aldrich, Burlington, MA), 60 U/ml DNase1 (Cat#18047019,Invitrogen, Carlsbad, CA), and 450 U/ml collagenase type I (Cat#C0130, Sigma-Aldrich, Burlington, MA), for 1 h at 37 °C with gentle rotation. The digested solution was vortexed for 20 s, flitted through a 40-μm nylon cell strainer (WHB, Shanghai, China) and topped up to 10 ml with Hank's Balanced Salt Solution (HBSS, Cat#AAPR25-1, PythonBio, Guangzhou, China) containing 2% fetal bovine serum (FBS, Cat#A3160802, Gibco, South American) and 0.2% BSA (Cat#AAPR615, PythonBio, Guangzhou, China). The cells were pelleted by centrifugation at 400 rcf for 5 min (4 °C) and washed in 1 ml Stain Buffer (FBS) (Cat#554656, BD Biosciences, San Jose, CA). After a second centrifugation (400 ref, 5 min, 4 °C), the cell pellet was resuspended in 100 μl stain buffer (FBS) containing 1% anti-CD16/CD32 (Cat#553141, BD Biosciences, San Jose, CA) and incubated for 15 min at room temperature prior to staining. For cell sorting, the prepared samples were incubated for 30–60 min on ice with the following antibodies from BD Biosciences (San Jose, CA): anti-CD45 APC-Cy7 (Cat#557659 1:100), anti-CD11b FITC (Cat#553310, 1:100), Ly-6G PE-Cy7 (Cat#560601, 1:100), anti-Ly-6C APC (Cat#560595, 1:40); or antibodies from Biolegend (San Diego, California): anti-CD45 BV605 (Cat#103137, 1:100), anti-CD11b FITC (Cat#101205), anti-F4/80 PE (Cat#123110), anti-Ly6G APC (Cat#127613), anti-MHCII APC/Cy7(Cat#107627), anti-CD163 BV421 (Cat#155309). The cells were sorted on a FACS Aria II cell sorter (BD Biosciences) directly into PBS for subsequent RNA isolation. At least 50,000 events were acquired and analyzed with FlowJo™ version 10.4.0 (Ashland, OR, USA: Becton, Dickinson and Company; 2019).

### Immunofluorescence

Infarcted hearts were fixed in 4% paraformaldehyde (PFA), embedded in paraffin, and sectioned at 5 μm intervals from the level of coronary artery ligation to the heart apex. After routine hydration, the heart section was microwaved for 3 min in citrate buffer (0.4 g/L citric acid and 3 g/L sodium citrate) for antigen retrieval. Permeabilization and blocking were performed in PBS with 5% bovine serum albumin (BSA) and 0.2% Triton X-100, at room temperature for 30 min. The sections were incubated overnight at 4 °C with the following primary antibodies: anti-HIMF (Cat#ab39626, Abcam, Cambridge, MA, USA), anti-CD68 (Cat#NBP-33337, Novus, CO, USA), anti-NOS2 (Cat#2D2-B2, R&D system, Minneapolis, MN), and anti-Arg1 (Cat#93668, Cell Signaling Technology, Danvers, MA, USA. Then the samples were incubated for 1 h at 30 °C with the following respective secondary antibodies: A-21070 (1:400), A-11006 (1:400), A11030 (1:400) (Invitrogen, Carlsbad, CA) and 4′,6–Diamidine–2′–phenylindole dihydrochloride (DAPI, Cat#10236276001, Sigma-Aldrich, Burlington, MA, USA). Confocal images were captured under a Zeiss LSM880 microscope (Carl Zeiss, Germany). A total of 3–4 animals were examined per group.

### Histological analysis of infarct size and collagen deposition

To assess the overall infarct extent, 5 μm paraffin sections of infarct heart tissue were prepared at five equal intervals from the ligation line to the heart apex, and stained with Masson trichrome (Cat#G1345, Solarbio, Beijing, China) following the manufacturer’s protocol. The percentage of the blue-stained area versus the total area was used to indicate the extent of the infarction. To assess the collagen deposition, three paraffin sections per heart containing the infarct region were randomly selected and stained with Picrosirius red (Cat#36324ES60, Yeasen, shanghai, China) following the manufacturer’s protocol. The percentage of the red-stained area versus the total area was applied and used to indicate the collagen density. Data analysis was performed with Image-J software (NIH, USA). A total of 3–4 animals were examined per group.

### Cell culture, cytokine stimulation and inhibitor treatment

For isolation and culture of murine bone marrow-derived macrophages (BMDMs), C57BL/6 J male mice (6–8 weeks old) were sacrificed and the femur and tibia were isolated under sterile conditions. The marrow was collected by repeatedly flushing the bone cavities of the femur and tibia with ice-cold PBS. The solution was then filtered through a 40-µm nylon cell strainer (WHB, Shanghai, China) and centrifuged at 1000 rcf for 5 min. The marrow pellet was resuspended in DMEM containing 10 ng/mL M-CSF (Cat#NBP2-35165, Novus, Novus, CO, USA), 10% FBS (Cat#A3160802, Gibco, Carlsbad, CA, USA) and 1% penicillin/streptomycin (Cat#15140122, Invitrogen, Carlsbad, CA, USA), then seeded in 35 mm plates and cultured in a 37 ℃ cell incubator (5% carbon dioxide and 95% air). The plates were supplemented with equal amounts of fresh DMEM medium (10 ng/mL M-CSF, 10% FBS, 1% penicillin/streptomycin) on day 3, and washed with PBS on day 7 to remove non-adherent or dead cells. The adherent BMDMs were kept in complete growth medium (DMEM containing 10% FBS and 1% penicillin/streptomycin) and tested by M1 and M2 type cytokine stimulation. Here, recombinant murine IL-4 (10 ng/ml), IFNγ (5 ng/ml) and LPS (1 μg/ml; all from R&D systems, Minneapolis, MN, USA) were applied for 24 h to promote the M1 and M2 polarization of BMDMs. Then, qRT-PCR analysis of M1 and M2 maker genes (NOS2 and Arg1, respectively) was performed in the stimulated cell samples. Primary cultured BMDMs were used without passaging. To inhibit STAT3 activity, 50 μM STAT3-specific inhibitor S3I-201 (Cat# HY-15146, MCE, Monmouth Junction, NJ, USA) was administrated 8 h before ad-HIMF/ad-GFP adenoviral infection. Equal amounts of DMSO or H_2_O were added as necessary and served as carrier controls.

The murine macrophage RAW264.7 cells were purchased from the Cell Bank of Chinese Academy of Sciences (Shanghai, China) and amplified though passaging. When ready to use, the RAW264.7 cells were grown in complete DMEM (supplemented with 10% FBS and 1% penicillin/streptomycin), and treated with cytokine stimuli or the STAT3 inhibitor S3I-201 as described above, when the cells reached ~ 60% confluence. The THP-1 cells were purchased from the Procell (Wuhan, China) and amplified though passaging. When ready to use, the THP-1 cells were grown in Roswell Park Memorial Institute (RPMI) 1640 medium (supplemented with 10% FBS, 1% penicillin/streptomycin and 0.05 mM β-mercaptoethanol), and treated with 100 ng/ml PMA (Phorbol 12-myristate 13-acetate, Cat# HY-18739, MCE, Shanghai, China) for 24 h to obtain adherent macrophages.

Cardiac fibroblast (CFs) were isolated from 1–2-day-old Sprague–Dawley (SD) rats, as previously described [15]. In brief, the hearts were removed from decapitated neonatal SD rats, immersed in PBS and minced with scissors into small pieces. The minced tissue fragments were digested in PBS containing 0.25% trypsin–EDTA at 37 °C and the isolated cells were added to fetal bovine serum (FBS) for subsequent centrifugation at 106 rcf for 5 min. The cells were resuspended in DMEM growth medium, and then pre-plated for 30 min at 37 °C to allow the fibroblasts to adhere to the plate. The cardiomyocytes were removed by changing the supernatant for fresh DMEM growth medium within 2 h after plating. Then, the CFs were allowed to recover and grow for 48 h in a 37 °C cell incubator prior to being seeded in experiment plates.

### Transfection of small interfering RNA

Small interfering (si)-HIMF, si-CHOP and the corresponding negative control RNAs were chemically synthesized by Ribobio (Guangzhou, China). The siRNA sense sequences designed were as follows: si-HIMF, 5′–GCACTAGTGTCAAGACTAT–3′; si-CHOP, 5′–GAAGAGCAAGGAAGAACTA–3′. Transient transfection of si-HIMF and si-CHOP into RAW264.7 and BMDM cells was accomplished with either Lipofectamine™ RNAiMAX Transfection Reagent (Cat#: 13778075, Invitrogen, Carlsbad, CA, USA) or Rfect siRNA/miRNA Transfection Reagent (Cat#:11013, Baidai biotechnology, Changzhou, China) according to manufacturer’s instructions. Macrophages were transfected with 50 nM siRNA and incubated for 48 h or 72 h before harvesting.

### Adenoviral transduction of macrophages

A green fluorescent protein (GFP)-tagged recombinant adenovirus encoding mouse HIMF (Ad-HIMF; Weizhen Biotech, Shandong, China) was used to induce controlled HIMF over-expression in BMDMs and RAW264.7 cells. A recombinant adenovirus-expressing GFP (Ad-GFP) was used as a control. BMDMs and RAW264.7 cells were infected with adenoviruses (MOI-30) for 48 h. Then, the DMEM culture medium was either replaced with fresh virus-free medium at 4 h after infection for analysis of the crosstalk between BMDMs or RAW264.7 cells and CFs, or not changed until harvesting.

### Cell death, migration, viability and proliferation analysis of CFs

To analyze the influence of HIMF expression on the crosstalk between macrophages and CFs, the culture media derived from HIMF-overexpressing or GFP-overexpressing BMDMs and RAW264.7 cells was used to grow CFs. brifely, the microphages were infected with ad-GFP or ad-HIMF virus for 4 h and washed with normal incubation medium, after another 48 h-incubation, the supernatants were harvested for the subsequent incubation of CFs. After 96 h incubation, the CFs were collected for subsequent function analyses. To detect cell death, CF slides were prepared for TUNEL assay as recommended (In Situ Cell Death Detection Kit, Roche, Basel, Switzerland). The percentage of dead cells was calculated as the number of TUNEL-stained cells (red) divided by number of DAPI-stained cells (blue) × 100%.

To analyze CF migration, a wound-healing assay was performed. Here, CFs were seeded in 6-well plates and grown to confluence, then driven into quiescence by culturing the cells in DMEM containing 0.5% FBS for 12 h. A line scratch was made in the monolayer of cells using a sterile p200 pipette tip. Photos of the scratch were taken immediately (0 h) and at 24 h after culturing under an Olympus inverted microscope. The speed of the cell migration was calculated as the mean linear movement of CFs over wound edges at 24 h, and expressed as a fold change compared with cells exposed to GFP-conditioned medium.

Cellular viability was measured according to the uptake of MTT ([4,5–dimethylthiazol–2–yl]–2,5-diphenyl tetrazolium bromide) (Sigma-Aldrich, Burlington, MA, USA). CFs were seeded in 96-well plates overnight and then treated with 110 μl DMEM-containing 0.5% FBS and 10 μl MTT solution (5 mg/ml). After incubation for a further 2 h, the MTT reaction were terminated by replacing the DMEM with 100 μl DMSO and subsequent shaking for 15 min. The absorbance was measured at 570 nm wavelength using an ELISA reader (Infinite 200 PRO, Tecan, Austria) and subtracted from that of a blank-containing medium buffer alone. The CF viability was defined as the relative absorbance of the treated versus the untreated control cells and expressed as fold change compared with cells treated with GFP conditioned medium.

The CF proliferation was determined using the colorimetric nonradioactive reagent bromodeoxyuridine (BrdU, Roche/ Sigma-Aldrich, Burlington, MA), following the manufacturer’s protocol. In brief, CFs were seeded and grown in 96-well plates overnight, starved in DMEM with 0.5% FBS for 24 h, then incubated with 10 μM BrdU-labeling solution for 24 h. After fixation for 30 min, the CFs were re-incubated with an HRP-coupled anti-BrdU-antibody for 90 min at room temperature, and 100 μL tetramethyl-benzidine solution was added for a further 5–30 min incubation until a blue color developed. The absorbance was measured at 370 nm (reference wavelength, 492 nm) in an ELISA plate reader and subtracted from that of a blank-containing medium buffer alone. CF proliferation was defined as the relative absorbance of treated versus untreated control cells, and expressed as fold change compared with cells treated with GFP conditioned medium.

### RNA reverse transcription and gene expression analysis

The total RNA from heart tissue and cell samples was extracted with TRIzol reagent (Invitrogen, Carlsbad, CA, USA) according to standard protocol, and quantified using a spectrophotometer at 260 nm. The quality was evaluated according to the A260/A280 and A260/A230 ratios (Molecular Devices, Holliston, MA, USA). Then, 500 ng–1 µg total RNA was treated with gDNA wiper mix (Vazyme, Nanjing, China) to remove genomic DNA contamination, and reverse transcribed using HiScript III qRT Supermix (Vazyme, Nanjing, China) according to the manufacturer’s instructions. For gene expression analysis, qRT-PCR was performed with ChamQ Universal SYBR qPCR master mix and gene-specific primers (Supplementary Table 1) on an QuantStudio 3 real-time PCR System (Applied Biosystems, Waltham, MA, USA). The expression level of each gene was normalized to internal control GAPDH gene and calculated using the 2^−∆∆Ct^ method.

### Western blot analysis

Heart tissues and cells were lyzed in RIPA lysis buffer (Solarbio, Beijing, China) supplemented with a protease inhibitor cocktail (Cat#HY-K0010, MCE, Monmouth Junction, NJ) and Phosphatase Inhibitor Cocktail II&III (Cat#HY-K0022& HY-K0023, all from MCE, Monmouth Junction, NJ, USA). After centrifugation at 15,294 rcf, 4 °C for 20 min, the supernatant was analyzed by BCA assay (Pierce, Thermo Scientific, Rockford, IL) to quantify the protein concentration. SDS-PAGE was performed by loading equal amounts of the proteins into the gel, which were then transferred to PVDF membranes (Merck Millipore, Bedford, MA, USA). The membrane was blocked with TBST solution containing 5% BSA. The target proteins were incubated overnight at 4 °C with the corresponding primary antibodies (Supplementary Table 2). Then horseradish peroxidase-conjugated anti-Rabbit (Cat#7074) or anti-Mouse (Cat#7076) secondary antibodies were used (Cell Signaling Technology, Danvers, MA). The blots were developed using Western Chemiluminescent HRP Substrate (Merck Millipore, Bedford, MA) and visualized using a UVP ChemStudio PLUS imaging system (CA, USA). To analyze protein phosphorylation, the membrane was stripped with Restore Western Blot Stripping Buffer (Thermo Scientific, Rockford, IL) and subsequently re-incubated with primary antibodies targeting the same position. Relative protein expression quantification was performed with Image-J software (NIH, USA).

### Statistical analyses

All data were analyzed using GraphPad Prism 8 software (CA, USA), and the results are expressed as the means ± Standard Deviation. A Student’s *t* test or Mann–Whitney test (*n* = 3 or 4 per group) was used for a comparison between two groups. A one-way ANOVA coupled with the Kruskal–Wallis test was used when analyzing more than two groups. A two-way ANOVA was used when analyzing two groups and ≥ 2 time points/treatments. Survival distributions were compared by the log-rank (Mantel–Cox) test. For all tests, a *p* < 0.05 was considered statistically significant.

## Results

### HIMF is upregulated after MI and exaggerates myocardium ischemic injury

**Fig. 1:**
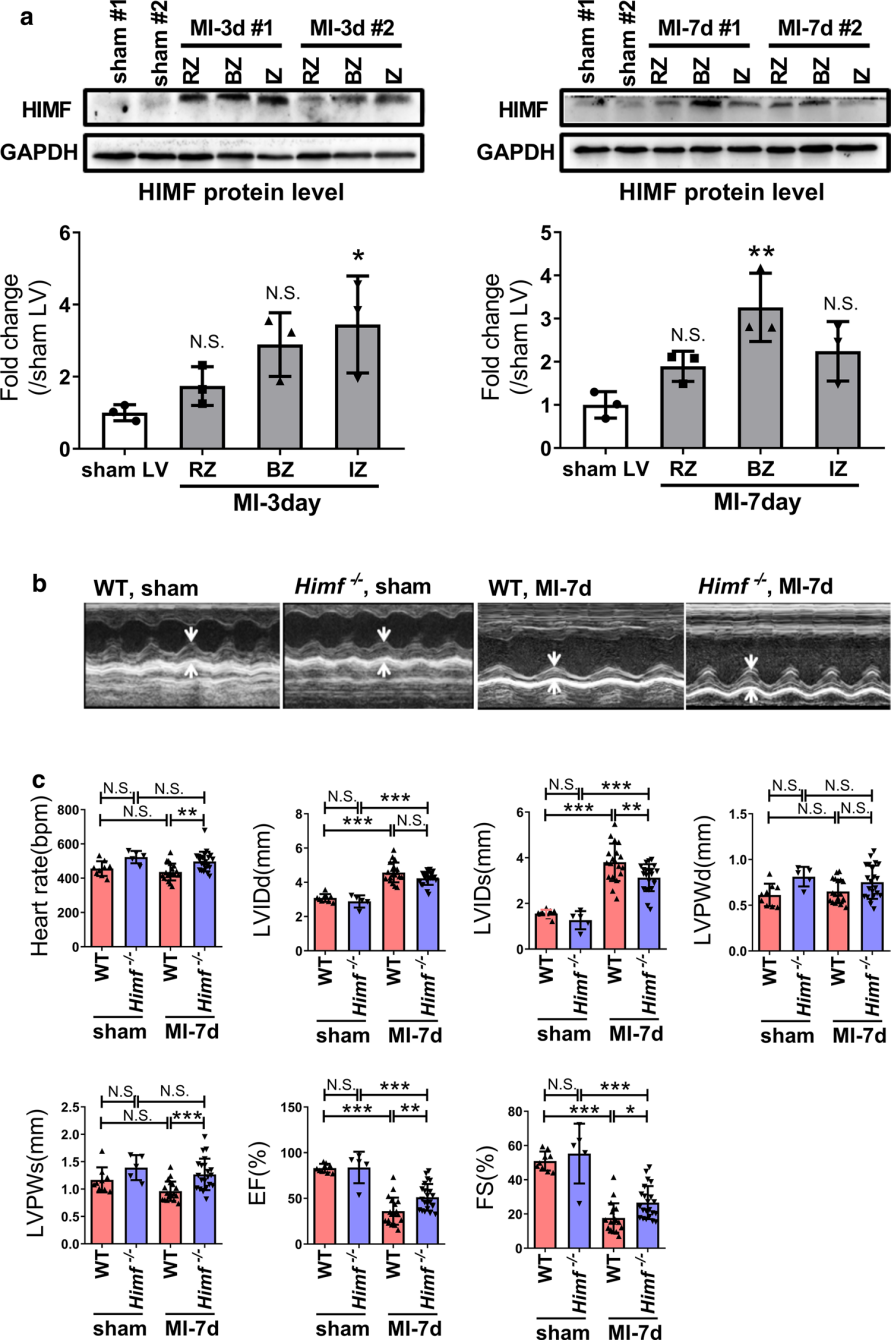
*Himf* ablation improves cardiac contractile function after MI. **a** Western blot analysis of HIMF protein levels in the left ventricle of sham and MI mice. Each left ventricle was divided into the infarct zone (IZ), border zone (BZ) and remote zone (RZ), according to the distance to the infarcted area. *n* = 3 mice per group. **b** Representative M-mode tracings from the echocardiography of wild type (WT) and *Himf*^*−/−*^ mice at 7 days after MI or sham operation. **c** Comparison of the echocardiographic parameters recorded for WT and *Himf*^*−/−*^ mice at day 7 after MI or sham operation. *LVIDs* systolic left ventricular internal dimension, *LVIDd* diastolic left ventricular internal dimension, *LVPWs* thickness of left ventricular posterior wall at systole, *LVPWd* thickness of left ventricular posterior wall at diastole, *EF* left ventricular ejection fraction, *FS* fraction shortening. *n* = 5–9 mice per group for sham control, *n* = 19–22 mice per group for MI operation. *p < 0.05, **p < 0.01, ***p < 0.001, *N.S.* not significant

HIMF expression was examined in the infarct zone (IZ), boarder zone (BZ) and remote zone (RZ) in the left ventricles of WT mice at 3 and 7 days post MI and WT sham-operated mice. HIMF protein level was significantly increased in IZ at 3 days post MI and in BZ at 7 days post MI (Fig. 1a). Then the cardiac response to MI was compared between WT and *Himf*^*−/−*^ mice. Echocardiography demonstrated a significant increase in the thickness of left ventricular posterior wall at systole (LVPWs) in *Himf*^−/−^ MI hearts compared with WT MI hearts (Fig. 1b, c). The left ventricular dilatation, evaluated by LVIDs, was significantly reduced in MI hearts from *Himf*^*−/−*^ mice compared to WT mice (Fig. 1c). Accordingly, the cardiac ejection fraction (EF) and fractional shortening (FS) were significantly higher in *Himf*^*−/−*^ mice than WT mice post MI (Fig. 1c), suggesting that an *Himf* deficiency improved the contractile performance of MI hearts.

**Fig. 2:**
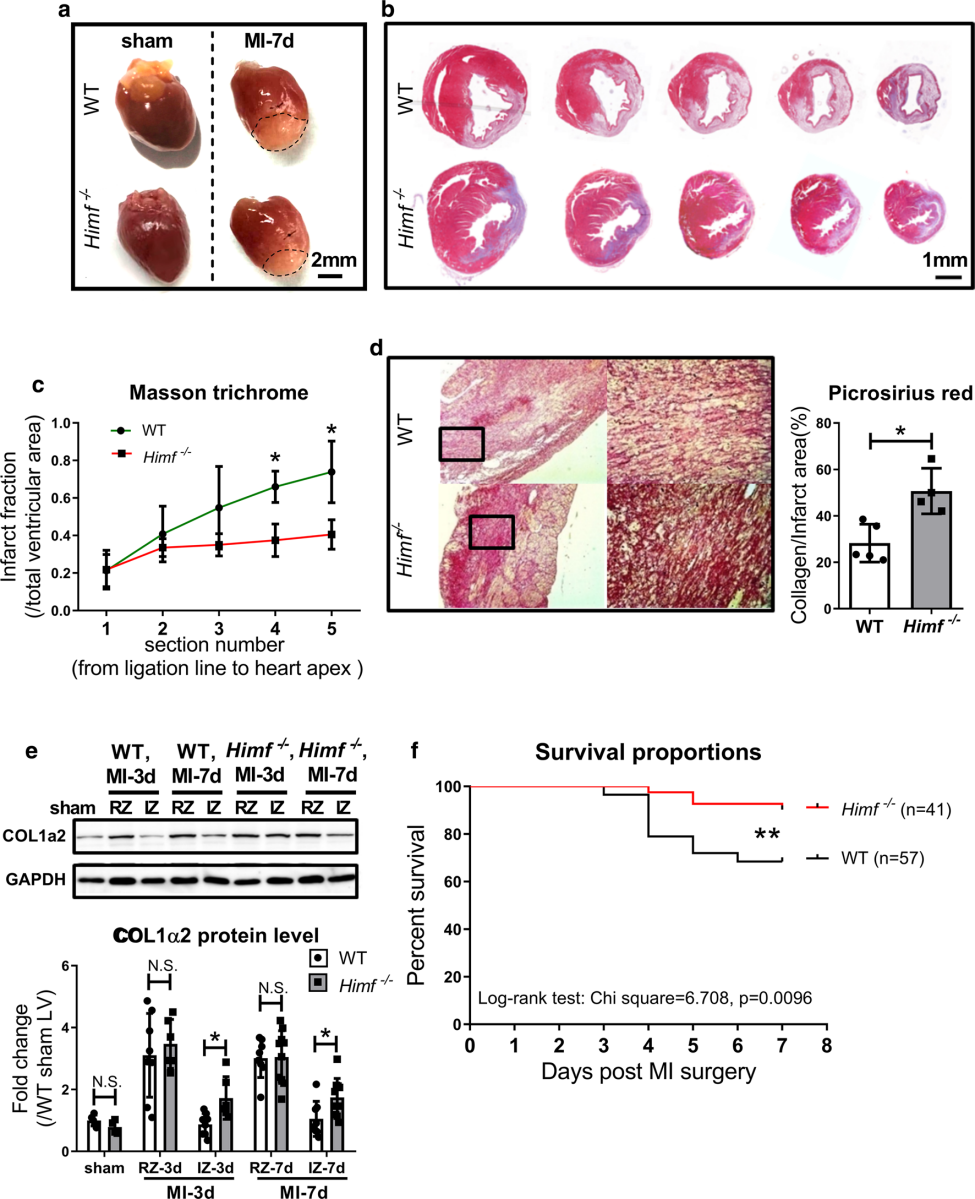
*Himf*^*−/−*^ mice exhibit a reduced infarct size, increased collagen fiber production and a higher survival rate in the 7 days after MI. **a** Representative images of WT and *Himf*^*−/−*^ hearts collected on day 7 after MI or sham operation. Scale bar = 2 mm. Sequential transverse sections of the MI hearts were prepared for Masson trichrome staining **b** and statistical analysis **c**. Scale bar = 1 mm. *n* = 3 mice per group. **d** Picrosirius red stain and statistical analysis of collagen production around the infarct region. *n* = 3–4 mice per group. **e** Western blot analysis of COL1α2 protein levels in the remote zone (RZ) and the infarct zone (IZ) of infarct hearts. *n* = 4–8 mice per group. **f** The rate of post-MI survival. *n* = 57 for WT mice, *n* = 41 for *Himf*^*−/−*^ mice. *p < 0.05, **p < 0.01, *N.S.* not significant

The infarct size in *Himf*^*−/−*^ hearts was observed smaller than in WT hearts at 7 days post MI (Fig. 2a). This was verified by Masson trichrome staining of transverse sections of the infarcted hearts (Fig. 2b, c). As adequate collagen deposition driven by cardiac fibroblasts (CFs) limits infarct expansion, picrosirius red (PSR)-staining was performed to examine the collagen deposition. The PSR results showed that the infarct region of *Himf*^*−/−*^ hearts was filled with denser collagen fibers (Fig. 2d). Consistently, type I collagen (COL1α2) protein expression in the infarct region of *Himf*^*−/−*^ hearts was found higher than that in WT hearts (Fig. 2e). Finally, we compared the survival rate between WT and *Himf*^*−/−*^ mice from day 1 to day 7 post MI. *Himf*^*−/−*^ mice displayed a significantly lower mortality rate than WT (Fig. 2f). Here, we observed that more WT mice died on day 3–5 post MI, likely due to observed cardiac rupture, than *Himf*^*−/−*^ mice. This is consistent with the report that exaggerated thinning of the LV wall and infarction expansion are often associated with cardiac rapture, a leading cause of mortality between day 3–5 post LAD coronary ligation [7].

### HIMF promotes M1 macrophage-mediated inflammatory response after MI

**Fig. 3:**
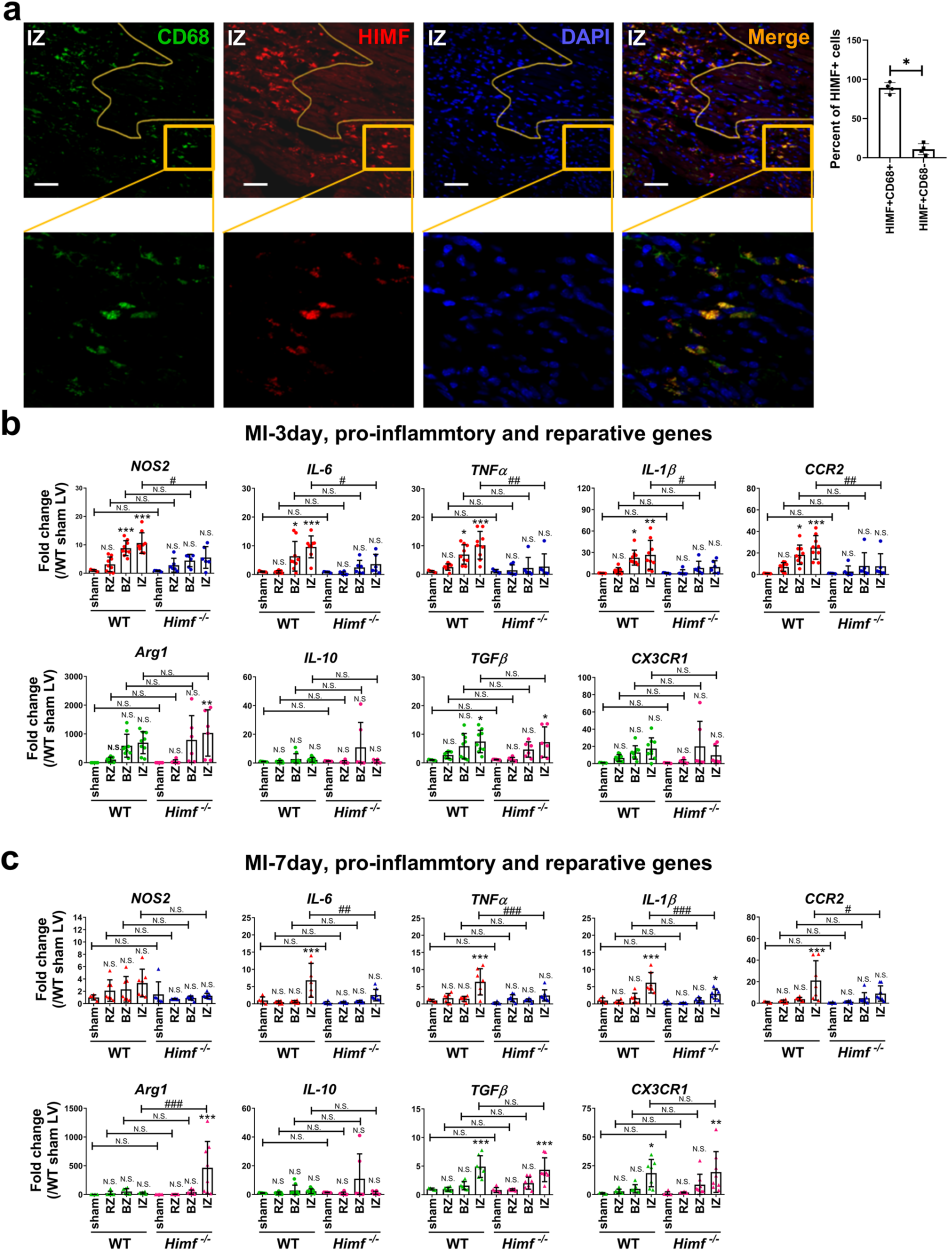
*Himf* deficiency results in a suppressed M1 inflammatory response in MI hearts. **a** Co-immunostaining of MI heart sections stained with the macrophage surface marker CD68 (green), HIMF (red) and DAPI (blue). The percentage of HIMF + CD68 + cells in the total HIMF + cells was calculated on the right. The yellow line indicates the boarder of the infarct region. *IZ* infarct zone. Scale bar = 50 μm. The lower panel represents a close-up of the staining, magnification = ×400. **b** mRNA expression analysis of pro-inflammatory and pro-reparative genes in the left ventricle collected on day 3 post-MI. For sham hearts, the left ventricles were collected as a control. For MI hearts, the infarct zone (IZ), border zone (BZ) and remote zone (RZ) were separately collected from each left ventricle. *n* = 5 mice for sham control, *n* = 6–9 mice for MI. **c** Same analysis was performed on day 7 post-MI. *n* = 5–6 mice for sham control, *n* = 7–8 mice for MI. *or # *p* < 0.05, ** or ## *p* < 0.01, *** or ### p < 0.001, *N.S.* not significant

Co-immunostaining for HIMF and the macrophage surface marker CD68 in transverse sections of the infarcted left ventricle identified higher HIMF protein levels in CD68 + cells than any surrounding area (Fig. 3a), suggesting that macrophages are a major source of HIMF production. The mRNA levels of M1-type inflammatory cytokines, including *IL-6*, *TNFα*, *IL-1β*, and the enzyme *NOS2* increased to milder extents in the BZ and IZ regions of *Himf*^*−/−*^ LV than WT LV at 3- and 7-day post MI (Fig. 3b, c). The M2-type reparative genes show different expression patterns. Namely the expression of *Arg1* (encodes an enzyme for collagen production) in the IZ region of the *Himf*^*−/−*^ left ventricle was significantly higher compared to WT at day 7 post MI (Fig. 3c). Except that, the mRNA levels of the anti-inflammatory M2 cytokines, including *IL-10*, *TGFβ*, *CX3CR1* were comparable in the BZ and IZ regions of *Himf*^*−/−*^ and WT hearts at 3- and 7-day post MI (Fig. 3b, c). These data suggest that HIMF ablation tempers the macrophage M1 pro-inflammatory response and facilitates M2 reparative activity.

Therefore, to examine if the improved healing outcome of *Himf*^*−/−*^ MI heart is due to the ablation of HIMF in macrophages, we constructed macrophage-specific knockout of HIMF mice (HIMF^flox/flox^; Lyz2-Cre (hereafter *Himf*-CKO, Supplementary Fig. 1). The hearts of *Himf*-CKO and HIMF-Floxed mice were collected at day 7 post MI (Supplementary Fig. 2a), and serial transverse sections were prepared for Masson trichrome staining. The results confirmed that ablation of HIMF in macrophage restricted infarct expansion (Supplementary Fig. 2b). Then the picrosirius red (PSR)-staining demonstrated enhanced collagen deposition around the infarct region of *Himf*-CKO hearts (Supplementary Fig. 2c). And the echocardiography demonstrated improved heart contractive performance in *Himf*-CKO mice (Supplementary Fig. 2d). These are consistent with the phenotypes observed in HIMF-full knockout mouse (*Himf*^*−/−*^), suggesting the macrophage-HIMF is a critical regulator of MI heart remodeling.

### *Himf* deficiency decreases M1-like but increases M2-like macrophage abundance after MI

**Fig. 4:**
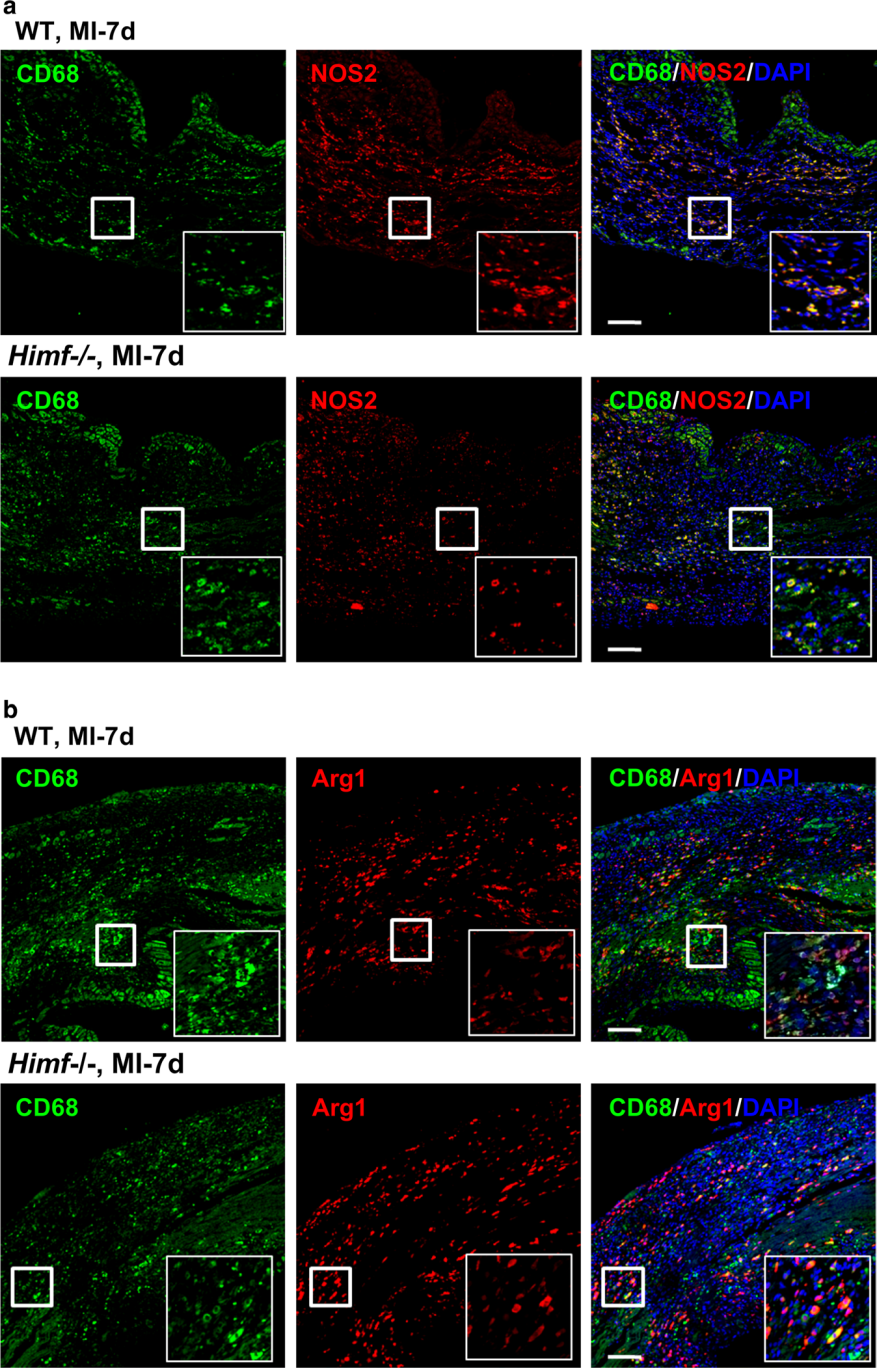

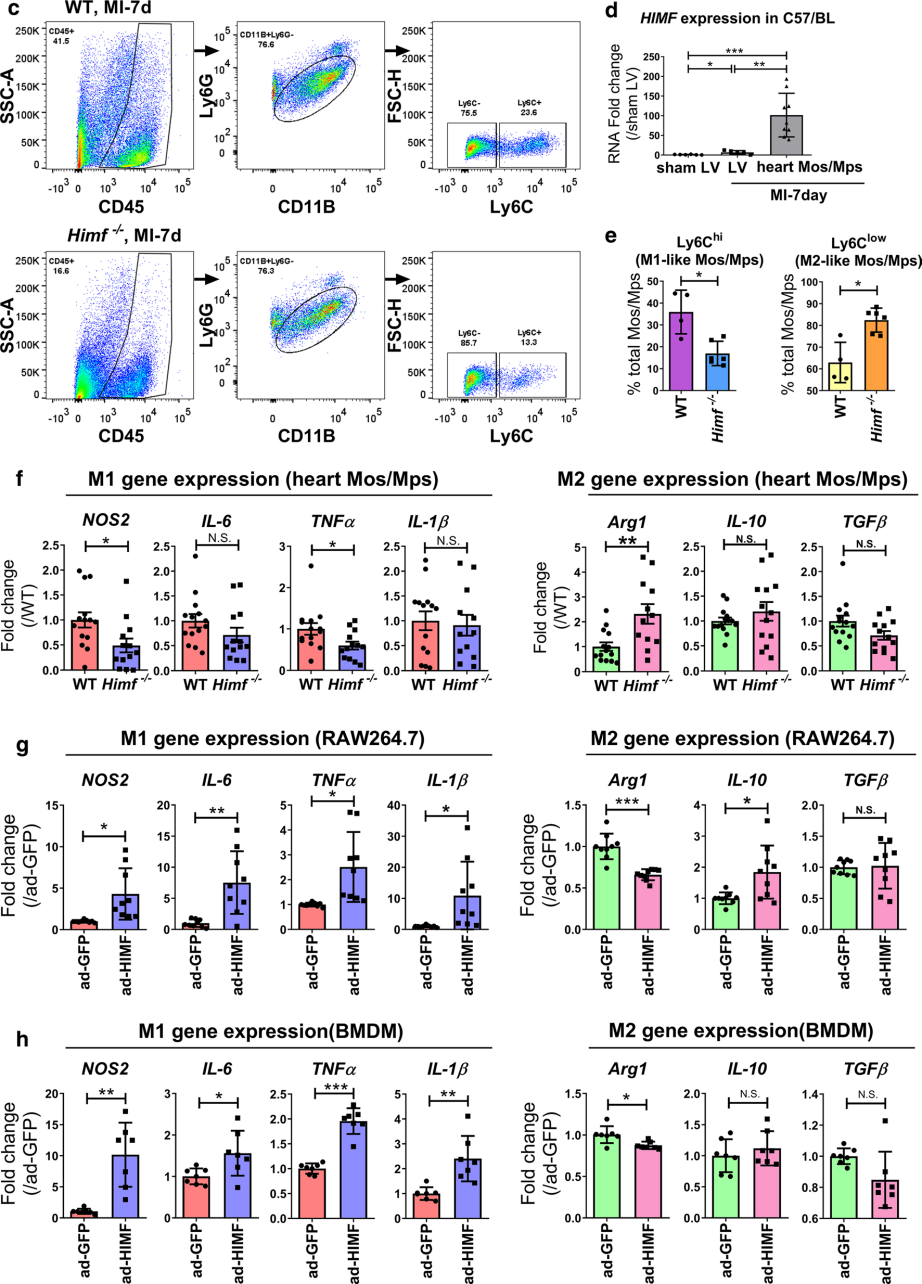
HIMF influences M1/M2-like macrophage polarization. **a** Co-immunostaining of CD68 (green) and NOS2 (M1 macrophage marker, red) in MI-heart sections from WT and *Himf*^*−/−*^ mice. Scale bar = 100 μM. White box: a close-up of staining, magnification = ×700. The nuclei counterstained with DAPI (blue). Transverse heart sections were prepared on day 7 post MI. **b** Co-immunostaining of CD68 and Arginase 1 (Arg1, M2 macrophage marker, red) in MI-heart sections from WT and *Himf*^*−/−*^ mice. **c** Flow cytometry sorting of M1-like and M2-like macrophage/monocytes from hearts on day 7 post MI. CD45 + CD11b + Ly6G + cells as shown in the middle panel were classified as neutrophiles, while the circled CD45 + CD11b+Ly6G-  cells were classified as monocytes/macrophage population (Mos/Mps). **d** Comparison of *Himf* mRNA expression in isolated heart macrophages/monocytes (CD45+CD11b+Ly6G-) and the homogenized left ventricle samples of MI and sham hearts. *n* = 5–9 mice per group. **e** The percentages of M1-like (CD45+CD11b+Ly6G-Ly6C+) and M2-like (CD45+CD11b+Ly6G-Ly6C-) cells sorted in **c**). *n* = 3–4 mice per group. **f** mRNA expression analysis of pro-inflammatory (left panel) and reparative (right panel) genes in macrophages/monocytes (CD45^+^CD11b^+^Ly6G^−^) sorted from hearts on day 7 post MI. *n* = 13–14 mice per group. **g**, **h** mRNA expression analysis of pro-inflammatory (left panel) and reparative (right panel) genes in RAW264.7 cells and bone marrow-derived macrophages (BMDMs) with or without adenoviral HIMF overexpression (ad-HIMF). A GFP-carrying (ad-GFP) adenovirus was used as an infection control. *n* = 7–9 replicates per group. *p < 0.05, **p < 0.01, ***p < 0.001, *N.S.* not significant

Pro-inflammatory NOS2 and tissue repair-related Arg1 are considered as functional markers of M1- and M2-type macrophages, respectively [4]. Co-immunofluorescence staining for NOS2/CD68, or Arg1/CD68 showed that the infarct region in the *Himf*^*−/−*^ LV had a smaller proportion of NOS2-expressing M1 macrophages, but a larger proportion of Arg1-expressing M2 macrophages than in the WT LV (Fig. 4a, b). To confirm this finding, M1/M2 macrophage populations were isolated from WT or *Himf*^*−/−*^ MI hearts by fluorescence-activated cell sorting (FACS). Based on established gating strategies [39, 44], isolated cells were differentiated into myeloid cells (CD45 + CD11b +), neutrophils (CD45 + CD11b + Ly6G +), and macrophages/monocytes (CD45 + CD11b + Ly6G −) (Fig. 4c). We found a much higher expression of *Himf* mRNA in isolated macrophages than whole heart tissues from WT mice after MI (Fig. 4d). Subgroups of macrophages/monocytes were further separated based on Ly6C + or Ly6C − as M1- or M2-type cells, respectively. Our quantifications showed that the M1-type macrophages/monocytes in *Himf*^*−/−*^ infarcted hearts accounted for < 20% of the total population, which is significantly lower than that in WT (~ 35%), although no significant difference in total number of macrophages was observed for two genotypes (Fig. 4c, e**)**. Supporting this, macrophages/monocytes population from infarcted *Himf*^*−/−*^ heart displayed a significant reduction in M1 marker gene (i.e. NOS2, TNFα) expression and an increase in M2 marker gene *Arg1* (Fig. 4f). To further verify the function of macrophage-HIMF in influencing M1/M2 transformation, we have performed the flow cytometry analysis on *Himf*-CKO mice and HIMF-floxed mice at day 7 post MI. With alternative gating methods, we also identified a significantly decreased portion of M1 macrophages (CD45 + CD11b + F4/80 + Ly6G–MHCII + CD163 −) and increased portion of M2 macrophages (CD45 + CD11b + F4/80 + Ly6G–MHCII–CD163 +) in the absence of macrophage-HIMF (Supplementary Fig. 3). We thus conclude that an *Himf* deficiency suppresses macrophage polarization into M1-like cells, and promotes the transition to a M2-dominant type following MI.

### HIMF promotes M1 inflammatory gene expression in BMDMs and RAW264.7 cells

To investigate the direct effect of HIMF on macrophage transformation, we manipulated *Himf* expression in RAW264.7 macrophages and BMDMs (Supplementary Fig. 4a, b). Adenoviral-mediated HIMF overexpression significantly activated the expression of M1 marker genes in both RAW264.7 cells and BMDMs compared with GFP controls (Fig. 4g, h). By contrast, HIMF overexpression resulted in a decrease in *Arg1* (Fig. 4g, h). siRNA-mediated *Himf* down-expression in BMDMs and RAW264.7 cells decreased the mRNA level of M1 marker *NOS2* (Supplementary Fig. 4c, d), mirroring the effects of HIMF overexpression. These data indicate that HIMF directly promotes macrophage transformation to an M1 phenotype, which coincides with the expression of a series of pro-inflammatory genes and suppression of M2 transformation.

### HIMF expression in macrophages impaired cardiac fibroblast activity through a paracrine effect

**Fig. 5:**
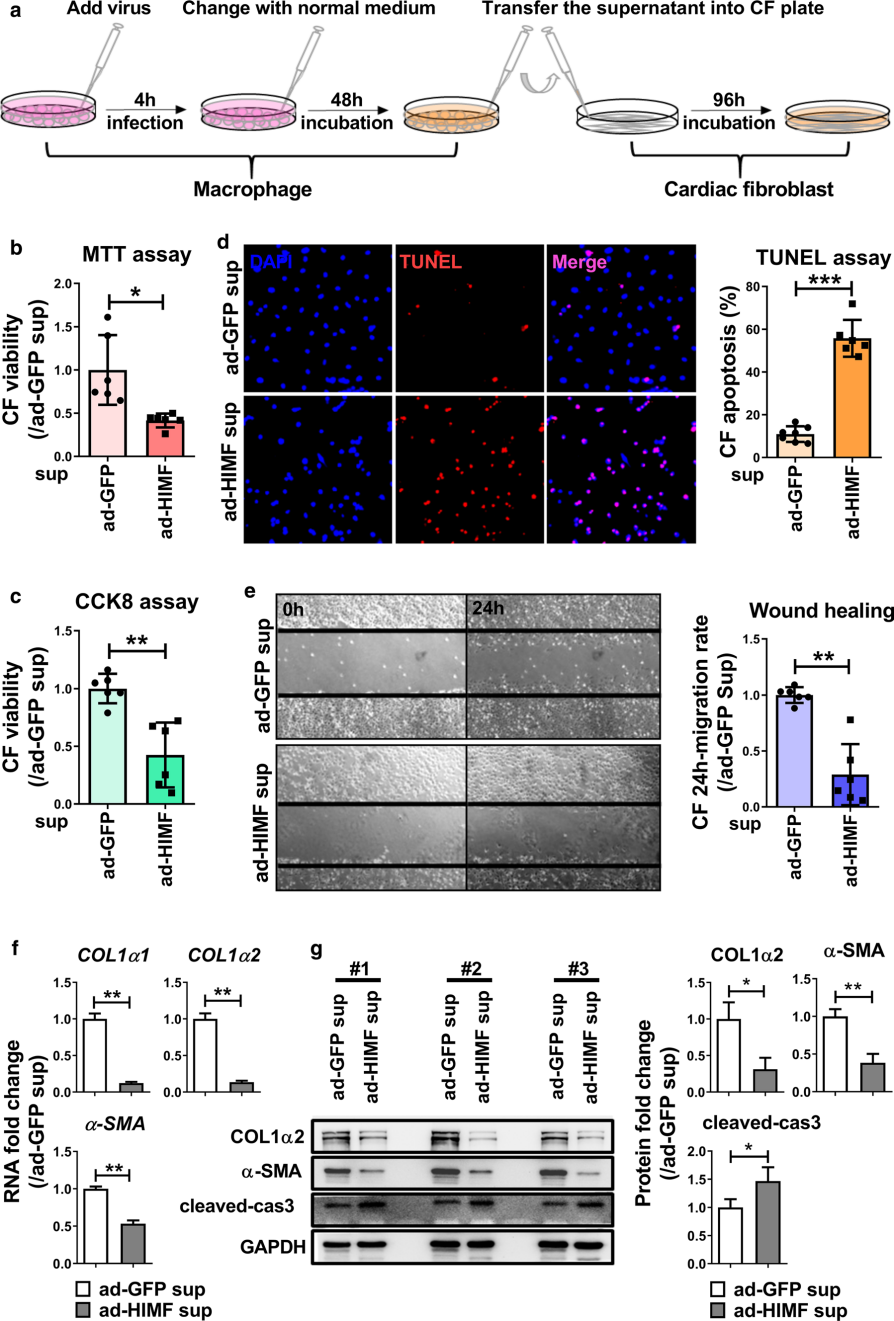
HIMF expression in macrophages impairs cardiac fibroblast (CF) function and promotes CF death. **a** The strategy to analyze the paracrine influence of macrophages on CFs. **b**, **c** Cell viability analysis of CFs by MTT and CCK8 assay. *n* = 6 replicates per group. ad-GFP sup: cultured with ad-GFP conditioned medium, ad-HIMF sup: cultured with ad-HIMF conditioned medium. **d** Cell death analysis of CFs by TUNEL assay. *n* = 6 replicates per group. **e** Migration analysis of CFs by wound healing assay. *n* = 6 replicates per group. **f** qRT-PCR analysis of *COL1α1*, *COL1α2* and α*-SMA* in CFs. *n* = 3 replicates per group. **g** Western blot analysis of COL1α2, α-SMA and cleaved-caspase 3 (cleaved-cas3) protein levels in CFs. *n* = 3 replicates per group. *p < 0.05, **p < 0.01, ***p < 0.001, *N.S.* not significant

*Himf* deficiency elevates collagen deposition in infarct region of hearts (Fig. 2d). However, *Himf* expression is not induced in cardiac fibroblasts (CFs) [15]. Hence, we explored the crosstalk between macrophages overexpressing HIMF and CFs. CFs were cultured in conditioned medium derived from macrophages overexpressing HIMF (ad-HIMF sup, Fig. 5a). MTT and CCK8 assays demonstrated that ad-HIMF medium impaired the CF viability (Fig. 5b, c). The level of the apoptosis marker cleaved-caspase 3 increased in CFs by ad-HIMF medium, and TUNEL staining also indicated increased CF death (Fig. 5d, g). Wound healing assay demonstrated that the ad-HIMF medium could inhibit CF migration (Fig. 5e). Meanwhile, ad-HIMF medium significantly decreased the mRNA and protein levels of α-SMA and COL1α2 in CFs (Fig. 5f, g). Similar results were obtained in RAW264.7 cells (Supplementary Fig. 5). These data implicate HIMF expression in macrophages impairs CF function, and thus myocardium wound healing.

### HIMF regulates macrophage polarization by activating CHOP expression

**Fig. 6:**
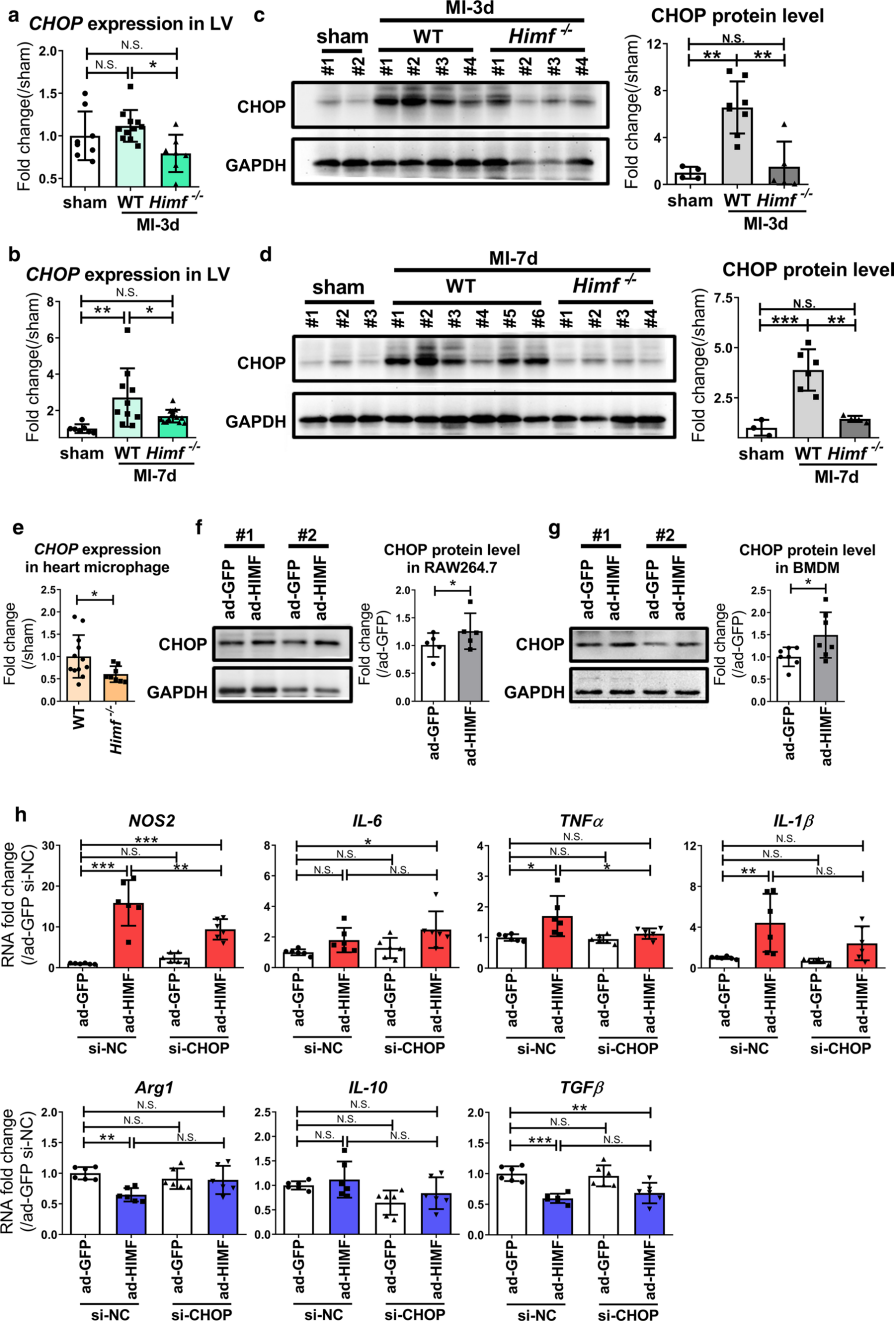
HIMF promotion of M1 macrophage polarization is partially mediated by CHOP. **a**, **b** *CHOP* mRNA expression analysis in infarct regions of WT and *Himf*^*−/−*^ hearts collected on day 3 and 7, respectively, after MI. For sham hearts, the left ventricle was collected on day 3 and 7, respectively, after sham operation. *N* = 7–11 mice per group. **c, d** Western blot analysis of CHOP protein levels in the infarct regions of WT and *Himf*^*−/−*^ hearts collected on day 3 and 7 after MI, respectively. *n* = 3–6 mice per group. **e** *CHOP* mRNA expression analysis in macrophages/monocytes from WT and *Himf*^*−/−*^ hearts. Heart macrophages/monocytes (CD45^+^CD11b^+^Ly6G^−^) were sorted by flow cytometry. *n* = 8–12 mice per group. **f**, **g** Western blot analysis of CHOP protein levels in RAW264.7 (*n* = 4) and BMDM cells (*n* = 7). **h** BMDM cells were transfected with an siRNA against *CHOP* (si-CHOP) or a negative control (si-NC) for 24 h, and then infected with ad-HIMF or ad-GFP for 48 h. The cells were harvested and the RNA expression levels of inflammatory (upper panel) and reparative (lower panel) genes were determined. *n* = 6 replicates per group. *p < 0.05, **p < 0.01, ***p < 0.001, *N.S.* not significant

Previous studies have demonstrated a critical role for the cellular stress sensor, C/EBP-homologous protein (CHOP) in determining macrophage polarity [33, 38, 45]. We found that the CHOP mRNA and protein levels were upregulated in MI hearts of WT mice, and this upregulation was inhibited in *Himf*^*−/−*^ mice (Fig. 6a-e). HIMF overexpression directly increased CHOP expression in BMDM and RAW264.7 cells (Fig. 6f, g, and Supplementary Fig. 6a, b). Decreasing CHOP expression by siRNA partially suppressed HIMF-induced M1 inflammatory cytokine production (Fig. 6h), and the decreased *Arg1* expression (Fig. 6h). Therefore, CHOP seems to mediate HIMF-induced M1 macrophage transformation and participates in the suppression of M2 transformation.

### CHOP–STAT1/STAT3 signaling pathway mediates HIMF-induced M1 polarization

**Fig. 7:**
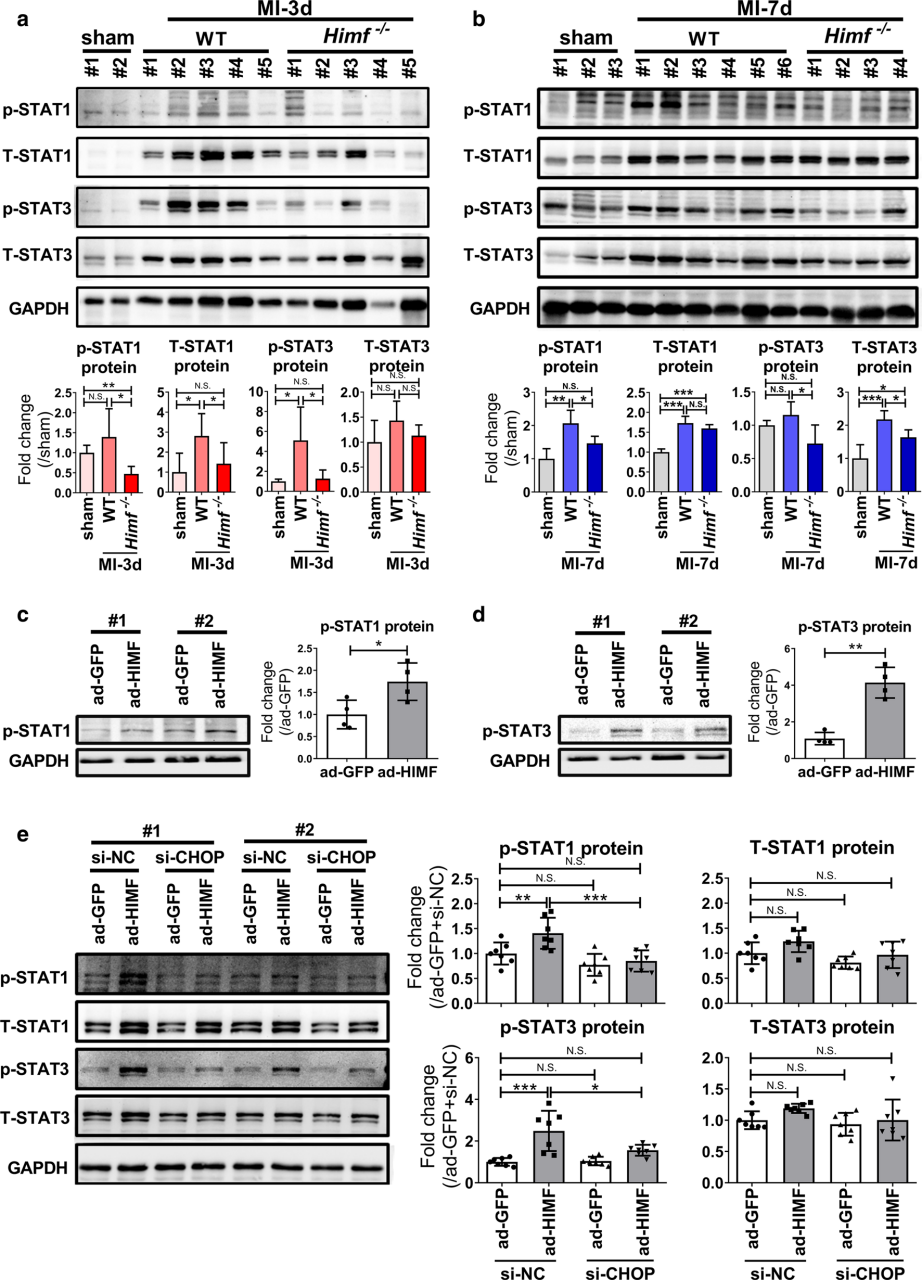
CHOP inhibition suppresses HIMF-mediated STAT1 and STAT3 phosphorylation. **a**, **b** Western blot analysis of phosphorylated STAT1 (p-STAT1), phosphorylated STAT3 (p-STAT3), total STAT1 (T-STAT1) and total STAT3 (T-STAT3) protein levels in the infarct regions of WT and *Himf*^*−/−*^ hearts collected on day 3 and 7, respectively, after MI. For sham hearts, the left ventricle was collected on day 3 and 7, respectively, after sham operation. *n* = 3–8 mice per group. **c**, **d** Western blot analysis of p-STAT1 and p-STAT3 protein levels in BMDMs with or without HIMF overexpression. *n* = 4 replicates per group. **e** BMDMs were transfected with si-CHOP or si-NC for 24 h, then infected with ad-HIMF or ad-GFP for 48 h before harvesting for western blot analysis of p-STAT1, p-STAT3, T-STAT1 and T-STAT3 protein levels. *n* = 7 replicates per group. *p < 0.05, **p < 0.01, ***p < 0.001, *N.S.* not significant

Signal transducer of activator of transcription (STAT) 1 is a well-established driver of M1 polarization [16]. We found that STAT1 activation correlated with the changes in CHOP expression in infarcted hearts, where the STAT1 phosphorylation level was increased in MI hearts of WT mice at 3 days and 7 days post MI; while *Himf* deficiency inhibited this effect (Fig. 7a, b). Over-expressing HIMF in BMDM and RAW264.7 cells consistently increased both CHOP expression (Fig. 6f, g) and STAT1 phosphorylation (Fig. 7c). siRNA-mediated CHOP knockdown suppressed STAT1 activation upon HIMF expression (Fig. 7e), suggesting STAT1 is a downstream signal of CHOP mediating HIMF-induced M1 polarization.

HIMF can activate STAT3 [15], and STAT3 might also be associated with M1/M2 cytokine production [8, 46]. We found the STAT3 activation pattern was quite similar to that of STAT1 in heart tissues (Fig. 7a, b) and cultured macrophages (Fig. 7d). siRNA-mediated CHOP down-expression inhibited STAT3 phosphorylation in HIMF-overexpressing cells (Fig. 7e), and STAT3 inhibitor S3I-201 suppressed HIMF-induced pro-inflammatory cytokine expression in BMDMs (Supplementary Fig. 7a, b). Therefore, HIMF promotes M1 polarization at least partially through CHOP–STAT1/STAT3 signaling pathways.

## Discussion

Monocyte and macrophage numbers expand rapidly in the heart after acute MI, driving post-MI repair and long-term ventricular remodeling [17]. The initial infiltrated monocytes/macrophages transform from a pro-inflammatory phenotype to a reparative phenotype, coordinating clearance of necrotic tissue and scar deposition to prevent myocardial rupture and limit functional deterioration. Manipulations to facilitate this monocyte/macrophage transformation are highly valued, but designing an effective intervention is difficult because the mechanisms are largely unknown [17, 22]. Here, we have established a novel mechanism whereby HIMF negatively regulates M1-to-M2 transformation post MI. Specifically, HIMF is upregulated in macrophages post MI, promoting macrophage M1 polarization and pro-inflammatory cytokine production. HIMF ablation inhibits the M1 inflammatory response and facilitates M2 transformation and reparative function, resulting in enhanced collagen deposition and tissue repair, reduced infarct expansion, and improved cardiac function and survival post-MI.

HIMF is a M2 macrophage marker, but its expression could also be induced by M1 type stimuli (LPS) (Supplementary Fig. 8). We found that HIMF is upregulated in macrophages throughout the M1-dominated pro-inflammatory and M2-dominated reparative phases of MI. More interestingly, HIMF was found to promote macrophage transformation to a M1 phenotype. The in vivo data show that HIMF expression levels positively correlate with M1 pro-inflammatory cytokine production, and that an *Himf* deficiency decreases the M1-type population (Ly6C^high^) and pro-inflammatory cytokine production post MI. The in vitro data provide direct evidence showing that HIMF stimulates macrophage transformation to a M1 phenotype. These data collectively indicate the critical role of HIMF in promoting macrophage polarization into the M1-like subgroup, exaggerating the inflammatory response and tissue damage post MI. Of note, this M2-like transition had negligible effects on anti-inflammatory M2 marker genes, such as IL-10, CX3CR1; and the function of CD163 + M2 macrophage in MI heart is currently elusive, especially considering quite distinct roles in the context of different diseases and conditions [9, 12, 34, 42]. Here, we can only speculate that an *Himf* deficiency likely tunes macrophages to exhibit a more reparative-prone status mainly by upregulating *Arg1* expression.

One of the most prominent beneficial changes that we observed in *Himf*^*−/−*^ and *himf*-CKO mice after MI was enhanced scar deposition at the infarction site. Scar deposition indicates adaptive remodeling that is necessary to prevent myocardial rupture, limit infarct expansion and ameliorate functional deterioration following acute MI [31]. We found that an *Himf* deficiency increases collagen density and collagen fibril assembly in the infarct region. However, exogenously applied HIMF induces CF proliferation, migration and myofibroblast differentiation [15, 20], suggesting that the effect of HIMF deficiency on promoting scar deposition in MI hearts is not due to the direct effect of HIMF on CFs. Interestingly, we found that the conditioned medium derived from HIMF-overexpressing macrophages decreased CF vitality and inhibited CF activation. Therefore, we conclude that the cytotoxic effects of HIMF-induced macrophage M1 polarization on CFs is overwhelming during the early stages of MI. *Himf* ablation facilitates M2 macrophage transformation, promoting scar deposition and limiting infarct expansion. It is worth mentioning that the infiltration of monocytes/macrophages may also contribute to the altered inflammation/repair outcome in the MI hearts of *Himf*^*−/−*^ and *himf*-CKO mice. This possibility should not be excluded, as the blood cell counts were not analyzed in this study.

Recent studies discovered a novel pro-inflammatory function of cellular stress protein CHOP in adipose tissue macrophages, promoting M1 polarization in the context of high-fat-diet-induced metabolic disorders [38]. Here, we found that CHOP was upregulated in heart tissues and macrophages after MI, positively correlating with HIMF expression and macrophage M1 polarization. CHOP knock-down prohibited M1 pro-inflammatory cytokine production. Besides that, STAT1 and STAT3 activation can regulate macrophage M1/M2 polarization and inflammatory responses [16, 35]. We found STAT1 and STAT3 were activated in WT but not *Himf*^*−/−*^ hearts during MI. STAT1 and STAT3 were also activated upon HIMF overexpression in BMDM and RAW264.7 cells, while CHOP knock-down inhibited STAT1 and STAT3 activation and pro-inflammatory cytokine production. Therefore, we conclude that HIMF upregulates CHOP expression to drive macrophage M1 transformation and a pro-inflammatory response via activation of STAT1 and STAT3 signaling (Supplementary Fig. 9). In addition, STAT3 was reported to exert cardioprotective effects in the context of myocardial ischemic or ischemia/reperfusion injury, by actively involving in the signal transduction of remote ischemic preconditioning, upregulating the expression of cardioprotective genes or improving the mitochondrial function [3–6]. Here, we proved that STAT3 mediates HIMF-induced expression of pro-inflammatory genes, suggesting a detrimental role of STAT3 may exist and complex the MI repair outcome. Since our study demonstrated HIMF was predominantly expressed in macrophages after MI, and its pro-inflammatory property played a causal role in the reduced scar deposition and infarct expansion, we hypothesize that STAT3 in macrophages partially mediates the detrimental role of HIMF [10, 11, 18, 36].

Rodent HIMF has two human analogues: resistin and RELMβ. HIMF might be functionally more similar to human resistin because they share similar expression patterns [25]. Specifically, mouse HIMF and human resistin are expressed in myeloid cells and are highly inducible. Clinical investigations have demonstrated a positive correlation between the circulating levels of resistin and the risk of MI [29, 43]. Others have suggested that resistin has pro-inflammatory properties, as recombinant resistin can activate pro-inflammatory cytokine expression in human peripheral blood mononuclear cells and monocytic THP-1 cells [2]. For the translational purpose, we infected THP-1 cells with adenovirus overexpressing human resistin (ad-RETN, Supplementary Fig. 10). By comparing the expression of M1 and M2 genes, we found the increased expression of M1 proinflammatory genes (i.e. NOS2, TNFα, IL-1β) and decreased M2 reparative gene (i.e. IL-10) upon RETN expression. This supports resistin shares the function similarity with HIMF. We thus speculate that our findings on HIMF might be extended to resistin, and resistin might serve as a novel promising target for the treatment of MI. Since no such studies investigating whether the resistin is majorly expressed from macrophage of MI patients, the clinical significance of macrophage-resistin under the MI background wroth being further explored.

## Supplementary Information

Below is the link to the electronic supplementary material.

Supplementary file1 (DOCX 3466 KB)

## Data Availability

All data needed to evaluate the conclusions in the paper are present in the paper and/or the Supplementary Materials. Additional data related to this paper may be requested from the authors.
