# Supplementary material for: HIMF deletion ameliorates acute myocardial ischemic injury by promoting macrophage transformation to reparative subtype
Source: Basic Res Cardiol. 2021 Apr 23;116(1):30. doi: 10.1007/s00395-021-00867-7 (PMC8064941; doi:10.1007/s00395-021-00867-7)
Supplement: Supplementary file 1 — Supplementary file1 (DOCX 3466 KB) [file 395_2021_867_MOESM1_ESM.docx]

**HIMF deletion ameliorates acute** **myocardial ischemic injury by promoting macrophage transformation to reparative subtype**

**Yanjiao Li,^1,2^† Min Dong,^1,2^† Qing Wang,^1,2^ Santosh Kumar,^1,2^ Rui Zhang,^1,2^ Wanwen Cheng,^1,2^ Jiaqing Xiang,^1,2^ Gang Wang,^1,2^ Kunfu Ouyang,^3^ Ruxing Zhou,^1,2^ Yaohong Xie,^1,2^ Yishen Lu,^1,2^ Jing Yi,^1,2^ Haixia Duan,^1,2^ Jie Liu^1,2*^**

^1^ Guangdong Key Laboratory of Genome Stability and Human Disease Prevention, Department of Pathophysiology, Shenzhen University Health Science Center, Shenzhen, 518060, China

^2^ Guangdong Key Laboratory of Regional Immunity and Diseases, Department of Pathology, Shenzhen University Health Science Center, Shenzhen, 518060, China

^3^ Drug Discovery Center, State Key Laboratory of Chemical Oncogenomics, School of Chemical Biology and Biotechnology, Peking University, Shenzhen Graduate School, Shenzhen, 51055, China

* **corresponding author:**
Jie Liu, M.D., Ph.D.
Department of Pathophysiology
Shenzhen University Health Science Center

Shenzhen 518060, China
Tel: +86-755-8667-4633
Fax: +86-755-8667-1906
Email : [liuj@szu.edu.cn](mailto:liuj@szu.edu.cn)

ORCID: 0000-0001-5310-7520

† These authors contributed equally to this work.

**Supplementary Material**


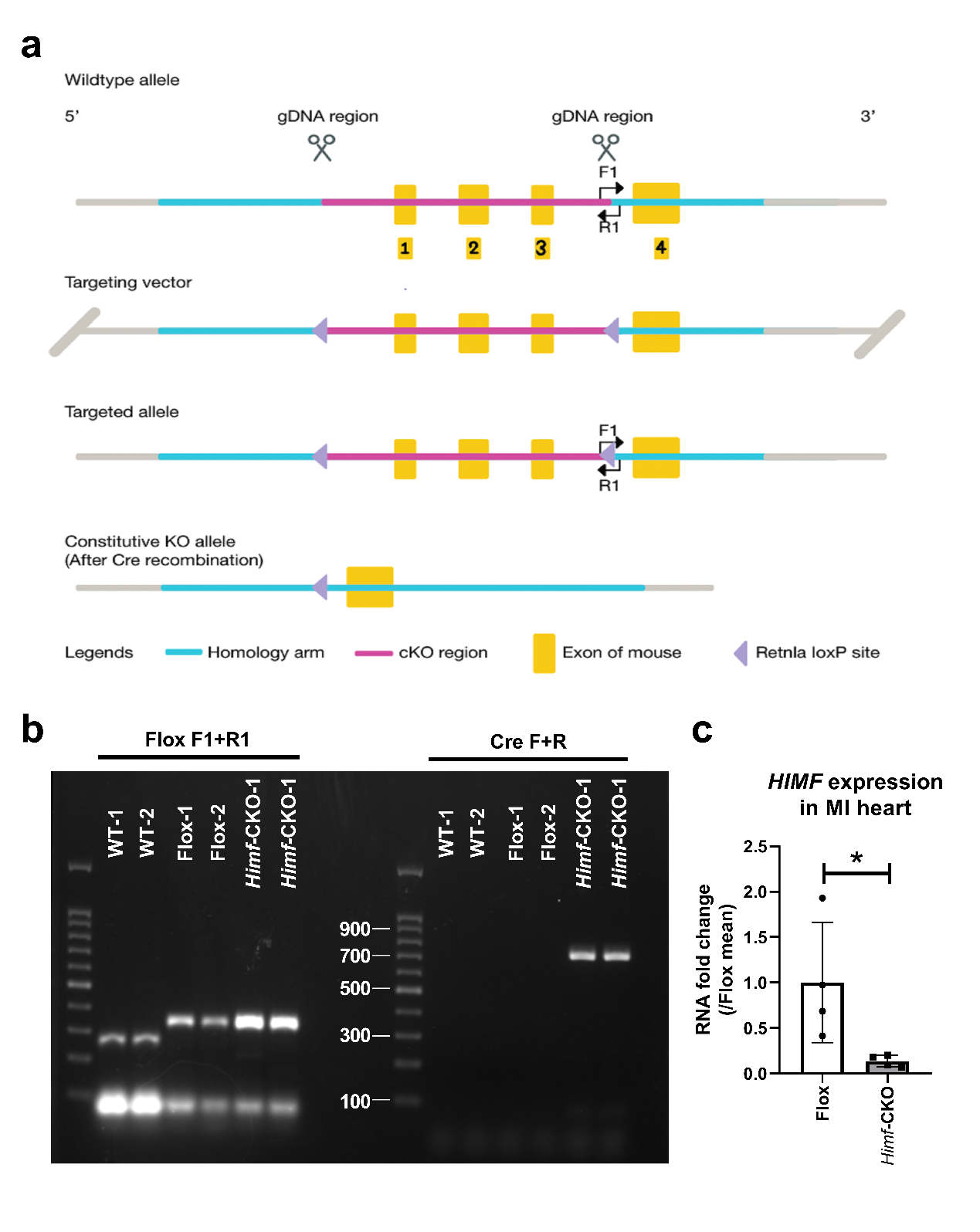
**Supplementary Fig. 1 Generation of macrophage specific HIMF knock-out mouse (HIMFflox/flox;Lyz2-Cre, or *Himf*-CKO). (a)** Strategy for constructing *Himf*-CKO mouse model. **(b)** Illustration of genotyping for Himf-CKO mice. 100bp DNA marker were loaded aside to indicate the size of PCR amplicons. **(c)** qRT-PCR analysis of HIMF expression in MI heart of *Himf*-CKO. The heart tissues were collected from both Flox and Himf-CKO mice at 7 day post MI. n = 4 mice per genotype. **p* < 0.05.


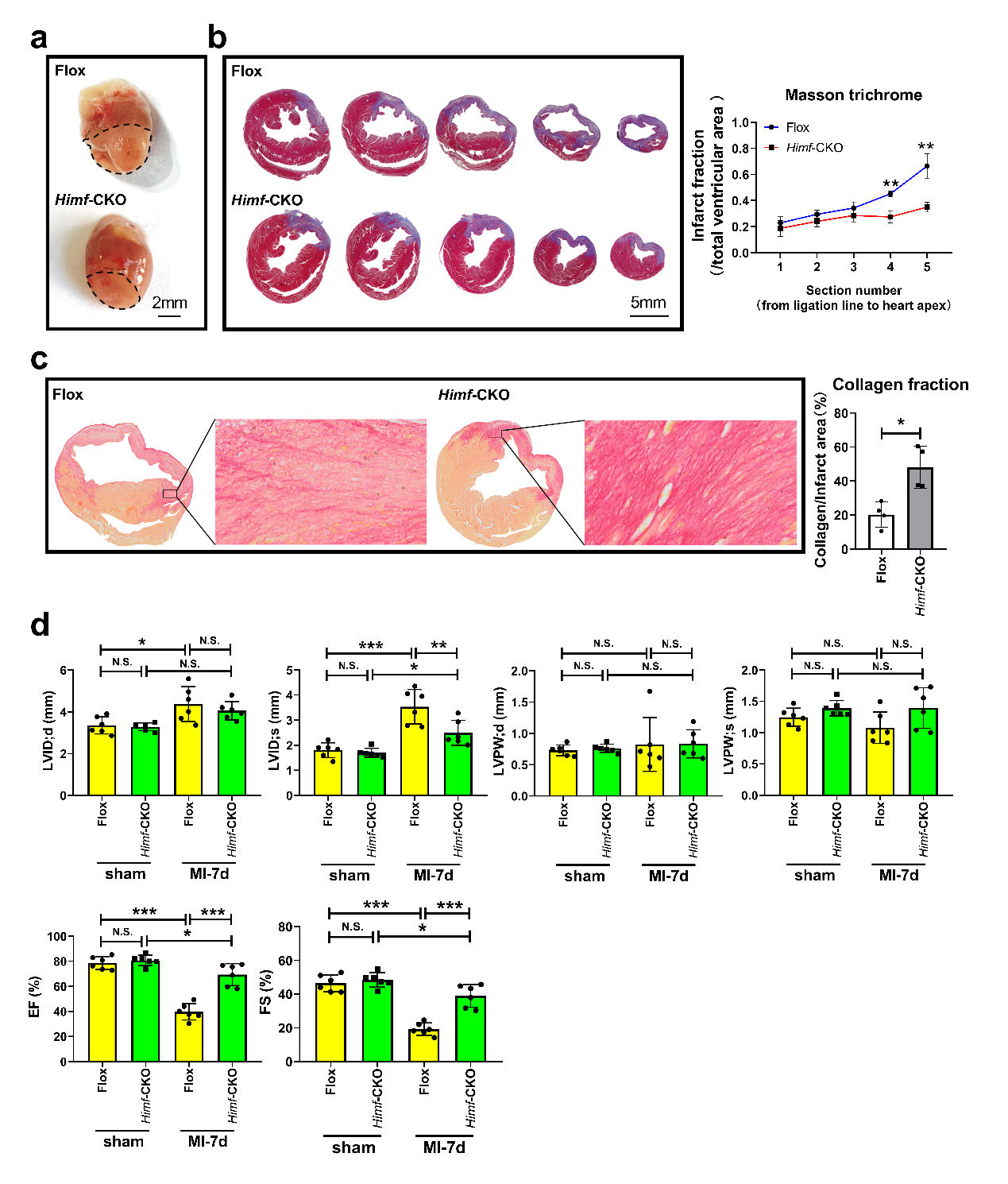


**Supplementary Fig. 2 *Himf-CKO* mice exhibit a reduced infarct size, increased collagen fiber production and improved LV contractile function. (a)** Representative images of HIMF-Floxed and *Himf*-CKO hearts collected on day 7 after MI operation. Dotted circle indicates the infarct zone. Scale bar = 2 mm. Flox: HIMF-Floxed mouse. **(b)** Masson trichrome staining and statistical analysis for sequential transverse sections of the MI hearts. Scale bar = 5 mm. n = 3 mice per genotype. **(c)** Picrosirius red stain and statistical analysis of collagen production around the infarct region. n = 4 mice per genotype. **(d)** Comparison of the echocardiographic analysis for HIMF-Floxed and *Himf*-CKO mice at day 7 after MI or sham operation. n = 6 mice per group. **p* < 0.05, ***p* < 0.01, ****p* < 0.001, N.S.: not significant.


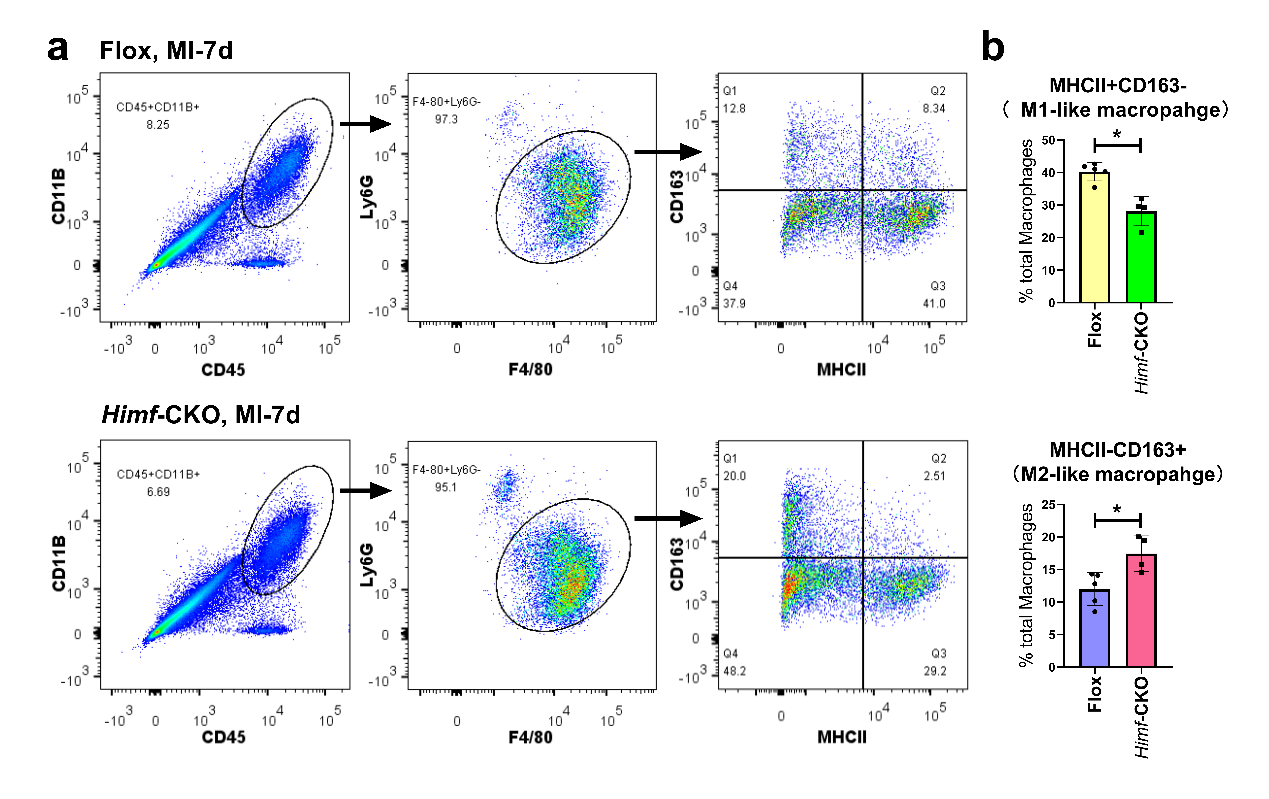


**Supplementary Fig. 3** **Macrophage-specific knockout of HIMF promotes M2 macrophage transformation. (a)** Representative image illustrating flow cytometry sorting of M1-like and M2-like macrophage from HIMF-floxed and *Himf*-CKO hearts on day 7 post MI. CD45^+^CD11b^+^ Cells were gated as myeloid cells (left panel), from which the F4/80^+^Ly6G^-^ cells were gated as macrophages (middle panel), and MHCII+CD163- macrophages were gated as M1 macrophages while MHCII-CD163+ were gated as M2 macrophages (right panel). **(b)** The percentages of M1-like macrophages (CD45+CD11b+F4/80+Ly6G-MHCII+CD163-) and M2-like macrophages (CD45+CD11b+F4/80+Ly6G-MHCII-CD163+) sorted in a). **p* < 0.05, ***p* < 0.01.


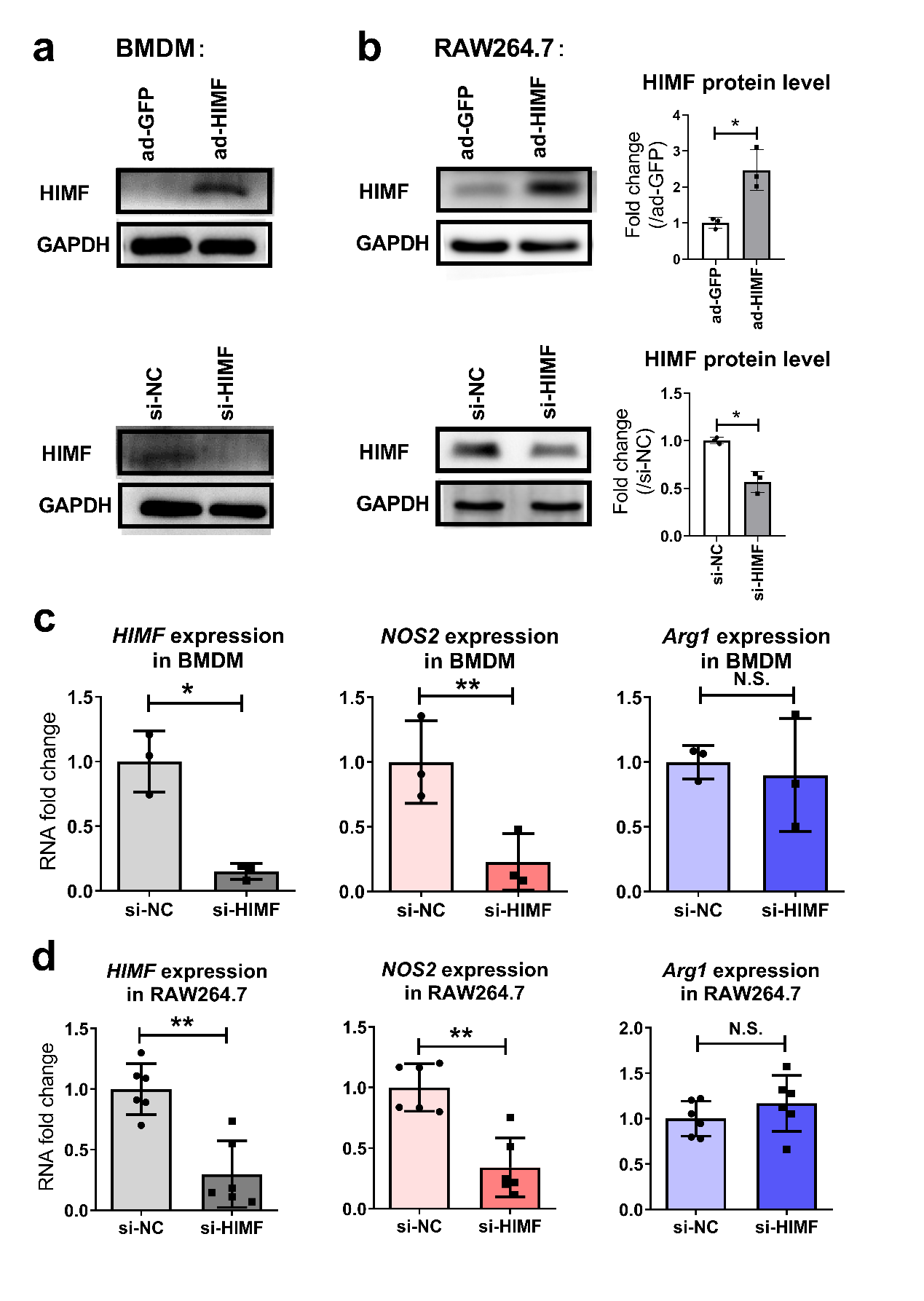
**Supplementary Fig. 4 Inhibiting HIMF expression results in decreased NOS2 expression.** **(a)** and **(b)** Western blot analysis of HIMF expression in BMDM and RAW264.7 cells with HIMF-overexpressing (ad-HIMF) or knockdown (si-HIMF). n=3 replicates per group. **(c)** and **(d)** mRNA expression analysis of *NOS2* and *Arg1* in BMDMs and RAW264.7 cells in *Himf*. For BMDMs, n = 3 replicates per group. For RAW264.7 cells, n = 6 replicates per group. **p* < 0.05, ***p* < 0.01, N.S.: not significant.


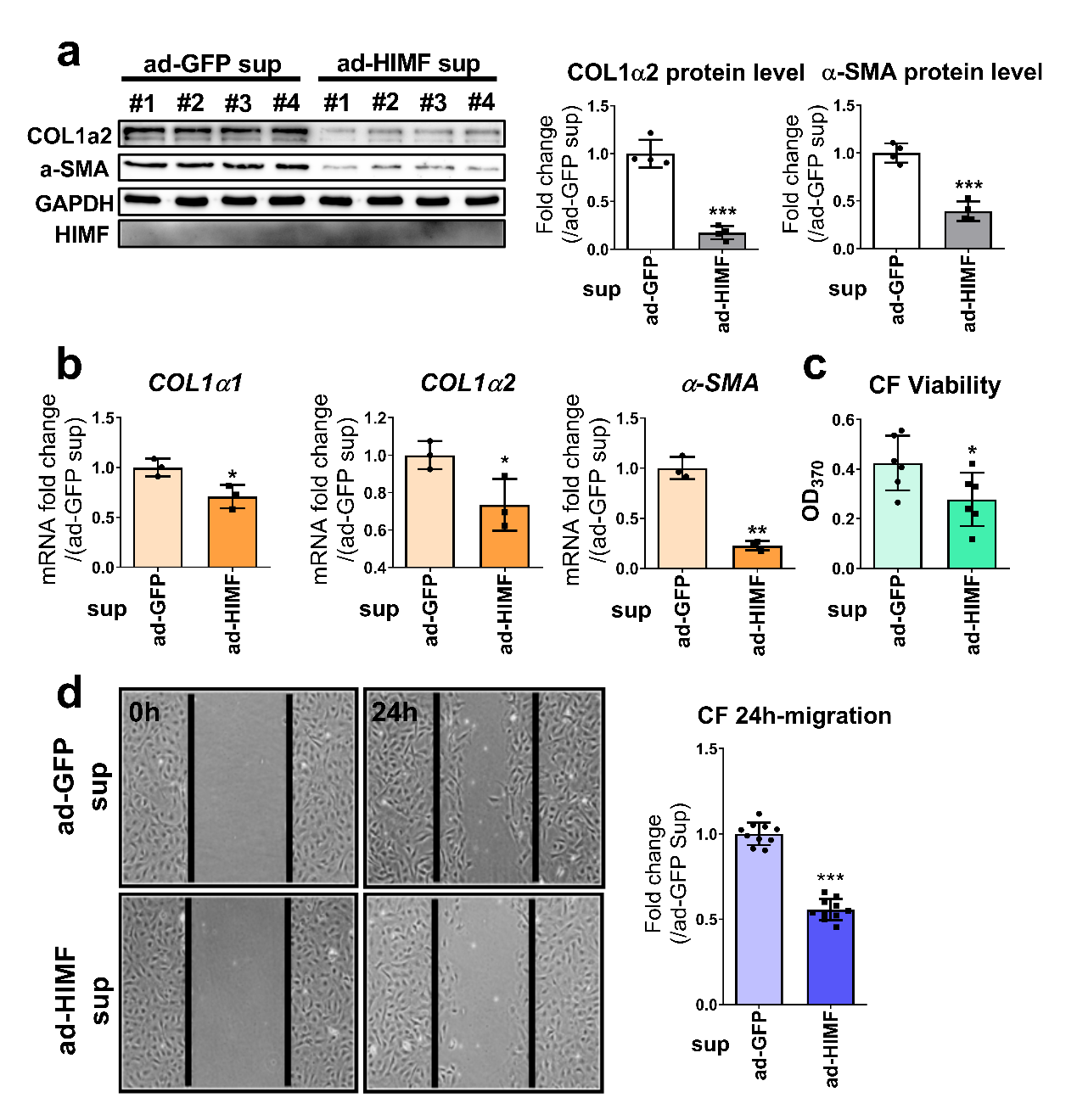


**Supplementary Fig. 5 HIMF expression in RAW264.7 cells impairs cardiac fibroblast (CF) function. (a)** Western blot analysis of COL1α2 and a-SMA protein levels in CFs incubated with ad-GFP or ad-HIMF conditioned medium. n = 4 replicates per group. **(b)** RNA expression analysis of COL1α1, COL1α2 and α-SMA in CFs incubated with ad-GFP or ad-HIMF conditioned medium. n = 3 replicates per group. The experimental strategy is illustrated in Figure 5A. **(c)** Cell viability analysis of CFs by BrdU assay. n = 6 replicates per group. **(d)** Migration analysis of CFs by wound healing assay. n = 6 replicates per group. **p* < 0.05, ***p* < 0.01, ****p* < 0.001, N.S.: not significant.


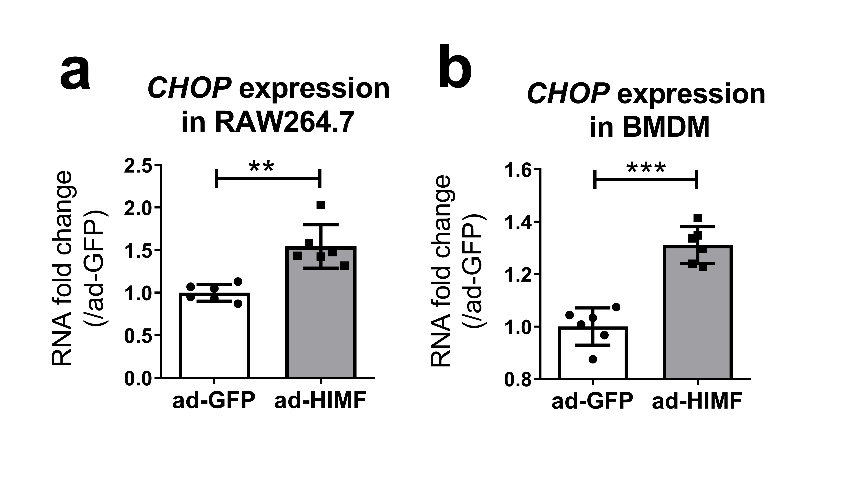


**Supplementary Fig. 6 HIMF-induced *CHOP* expression in macrophages**. *CHOP* mRNA expression levels in BMDMs **(a)** and RAW264.7 cells **(b)** with or without HIMF overexpression (ad-HIMF). n = 6 replicates per group for BMDMs and RAW264.7 cells. ***p* < 0.01, ****p* < 0.001, N.S.: not significant.


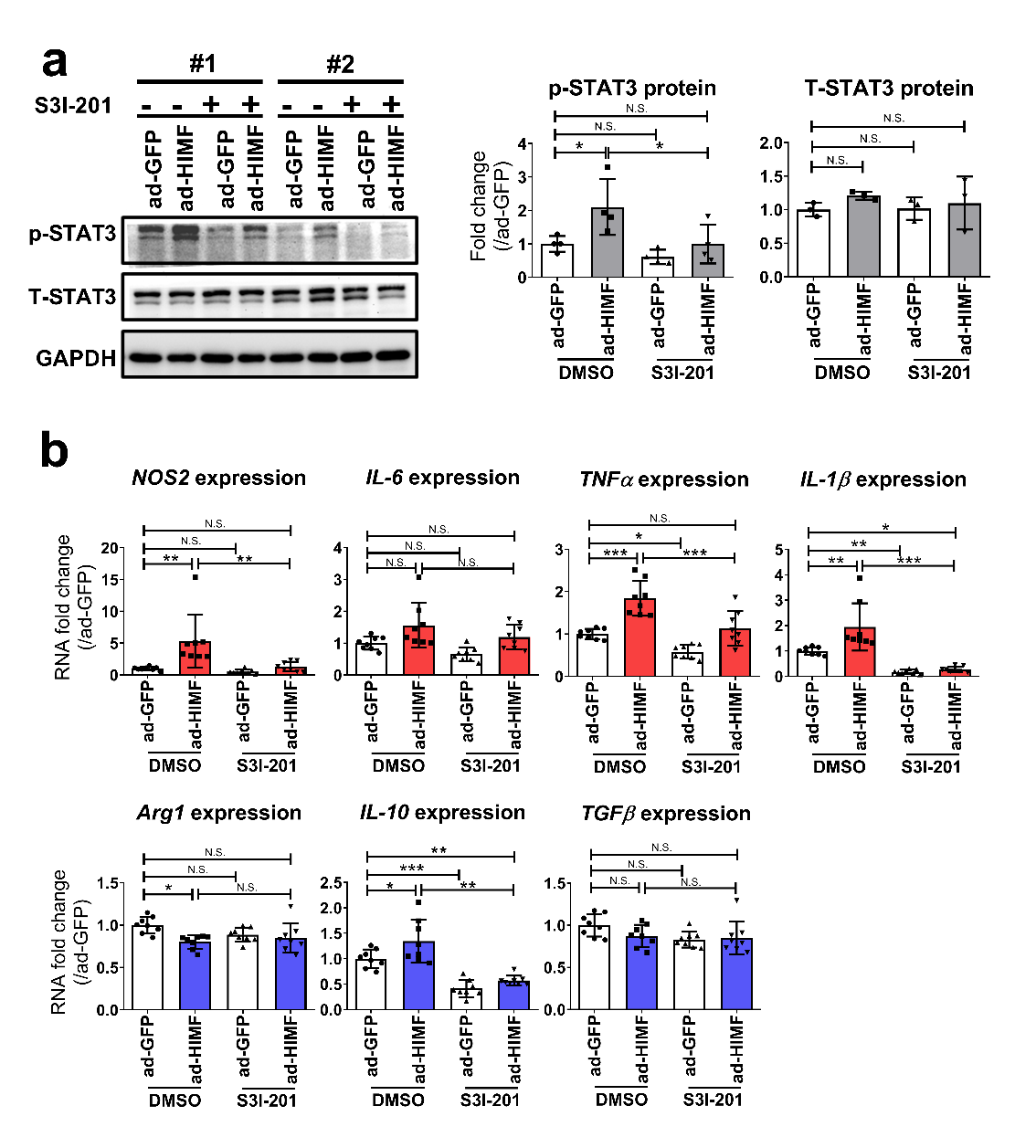


**Supplementary Fig. 7 STAT3 mediates HIMF pro-inflammatory effect. (a)** Western blot analysis of p-STAT3 and T-STAT3 with or with HIMF overexpression (ad-HIMF) following STAT3 inhibitor (S3I-201, 50 μM) treatment. n = 4 replicates per group. **(b)** BMDMs were treated with S3I-201 (50 μM)for 24 h, then infected with ad-HIMF or ad-GFP for 48 h before RNA expression analysis of inflammatory (upper panel) and reparative (lower panel) genes. n = 8 replicates per group. **p* < 0.05, ***p* < 0.01, ****p* < 0.001, N.S.: not significant.


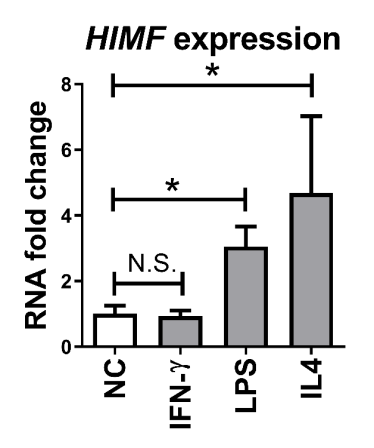


**Supplementary Fig. 8 HIMF expression was induced in LPS-treated macrophage**. RAW264.7 cells were treated with M1 stimuli (5 ng/ml IFN-γ, 1 μg/ml LPS) and an M2 stimulus (10 ng/ml IL4) for 24 h. The cells were harvested for *Himf* mRNA expression analysis. n = 3-8 per group. **p* < 0.05, N.S.: not significant.


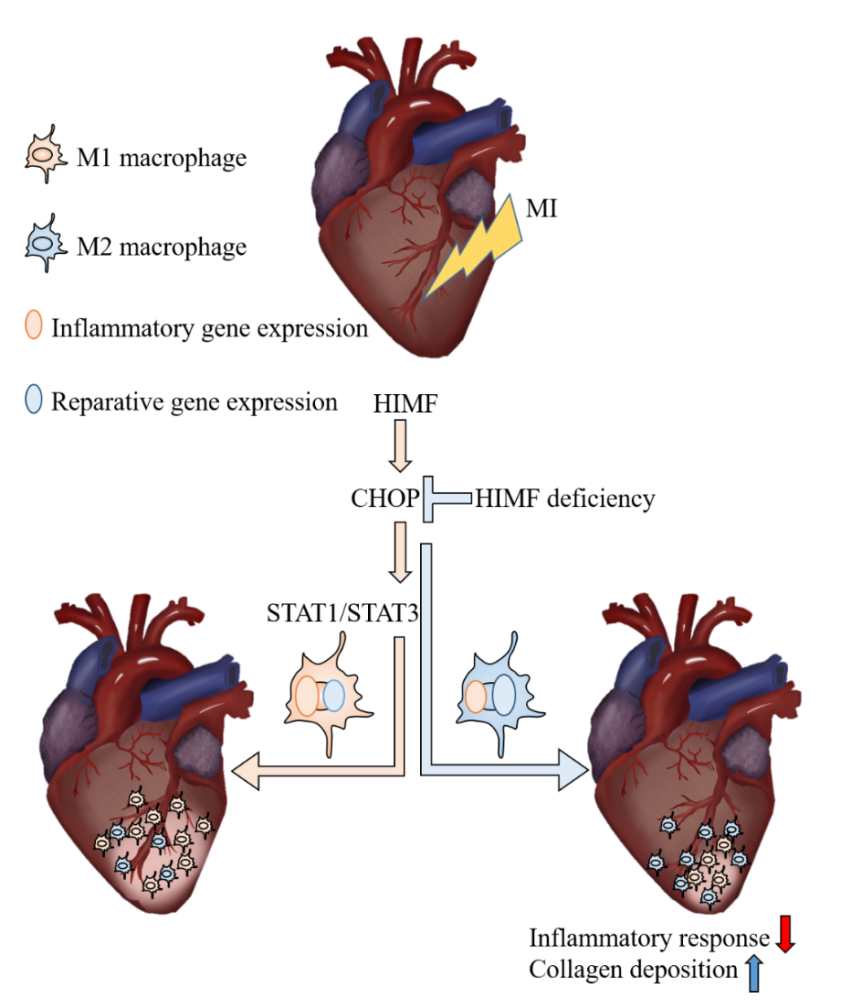


**Supplementary Fig. 9 HIMF deficiency ameliorates acute myocardial ischemic injury by promoting macrophage transformation to reparative subtype.** In the early inflammatory and reparative phases of MI, HIMF promotes M1-type macrophage polarization via inducing CHOP expression and STAT1/STAT3 activation. Deletion of HIMF reduces CHOP expression and facilitates the transition to M2-type macrophage, resulting in reduced inflammatory response and improved reparative effects.


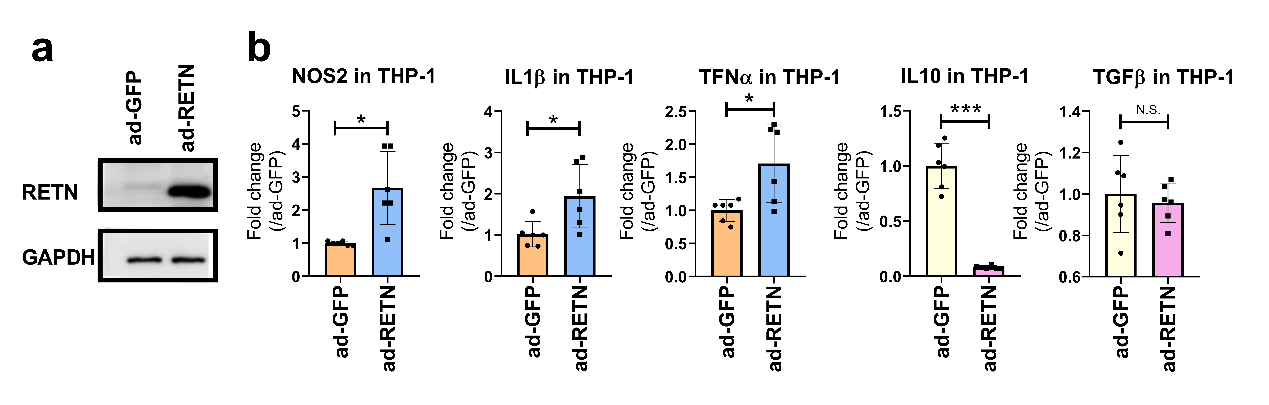


**Supplementary Fig. 10 Both human resistin and murine HIMF promote M1 gene expression in THP-1 cells .** **(a)** Western blot analysis of Resistin (RETN) expression in THP-1 cells overexpressing GFP (ad-GFP) or RETN (ad-RETN) . n=3 replicates per group. **(b)** mRNA expression analysis of M1/M2 genes in RETN-overexpressing THP-1 cells. n=6 replicates per group. **(c)** Western blot analysis of HIMF expression in THP-1 cells. n=3 replicates per group. **(d)** mRNA expression analysis of M1/M2 genes in HIMF-overexpressing THP-1 cells. **p* < 0.05, ***p* < 0.01, ****p* < 0.001, N.S.: not significant.

**Supplementary Table 1 primers for rea-time RT-PCR**

| Gene | Sense | Antisense |
| --- | --- | --- |
| MM-HIMF | AGGAACTTCTTGCCAATCCAGC | CACAAGCACACCCAGTAGCA |
| MM-NOS2 | GCACCGAGATTGGAGTTC | GAGCACAGCCACATTGAT |
| MM-IL-6 | TCCATCCAGTTGCCTTCTTG | GGTCTGTTGGGAGTGGTATC |
| MM-TNFα | TGCCTATGTCTCAGCCTCTTC | AGGCCATTTGGGAACTTCT |
| MM-IL-1β | TCGTGAATGAGCAGACAG | ATCAGAGGCAAGGAGGAA |
| MM-CCR2 | GTCACAGGATTAGGAAGGTT | GGTTCAGTCACGGCATAA |
| MM-Arg1 | AAGGTCTCTACATCACAGAAG | CGAAGCAAGCCAAGGTTA |
| MM-IL-10 | GAGCAGGTGAAGAGTGATT | TCCAGCAGACTCAATACAC |
| MM-TGFb | CAACAACGCCATCTATGAG | CAAGGTAACGCCAGGAAT |
| MM-CX3CR1 | TCATCCAGGTTCTATCATCAG | ATTCCAACAGCATCTTAGGT |
| RN-Col1α1 | CGAGTATGGAAGCGAAGG | GCAGTGATAGGTGATGTTCT |
| RN-Col1α2 | CCTGCCATTCCTTGACAT | CTCTGACCAATCCTTCTCTT |
| RN-α-SMA | AGAACACGGCATCATCAC | GTCCAGCACAATACCAGTT |
| MM-CHOP | CCTAGCTTGGCTGACAGAGG | CTGCTCCTTCTCCTTCATGC |
| MM-GAPDH | GGTTGTCTCCTGCGACTTCA | TGGTCCAGGGTTTCTTACTCC |

**Supplementary Table 2 primary antibodies for western blot analysis**

| Antibodies | Species | Dilution | Company | Catalogue No.# |
| --- | --- | --- | --- | --- |
| anti-HIMF | Rabbit | 1:1000 | Abcam | ab39626 |
| anti-GAPDH | Rabbit | 1:5000 | Cell Signaling Technology | 2118S |
| anti-Col1a2 | Rabbit | 1:2000 | Abclonal | A5786 |
| anti-a-SMA | Rabbit | 1:1000 | Cell Signaling Technology | 19245 |
| anti-cleaved-caspase 3 | Rabbit | 1:1000 | Cell Signaling Technology | 9664 |
| anti-CHOP | Mouse | 1:1000 | Cell Signaling Technology | 2895 |
| anti-p-STAT1 (S727) | Rabbit | 1:1000 | Abclonal | AP0109 |
| anti-STAT1 | Rabbit | 1:1000 | Abclonal | A19563 |
| anti-p-STAT3 (Y705) | Rabbit | 1:1000 | Cell Signaling Technology | 4904 |
| anti-STAT3 | Rabbit | 1:1000 | Cell Signaling Technology | 9145 |
